# Discovery of a new chemical scaffold for the treatment of superbug *Candida auris* infections

**DOI:** 10.1080/22221751.2023.2208687

**Published:** 2023-05-11

**Authors:** Jie Tu, Tianbao Zhu, Qingwen Wang, Wanzhen Yang, Yahui Huang, Defeng Xu, Na Liu, Chunquan Sheng

**Affiliations:** a School of Pharmacy, Second Military Medical University (Naval Medical University), Shanghai, People’s Republic of China; b School of Pharmacy, Changzhou University, Changzhou, People’s Republic of China

**Keywords:** *Candida auris*; benzoanilide; antifungal; drug resistance; *Candida albicans*

## Abstract

*Candida auris* has emerged as a serious threat of public health and caused global epidemic due to multi-drug resistance, remarkable transmissibility and high mortality. To tackle the challenging super fungus, novel benzoanilide antifungal agents were discovered by an integrated strategy of phenotypic screen, hit optimization, antifungal assays and mechanism exploration. The most promising compound **A1** showed potent *in vitro* and *in vivo* efficacy against *Candida auris* infection. Mechanism investigation revealed that compound **A1** blocked the biosynthesis of virulence factors and fungal cell walls through the inhibition of glycosylphosphatidylinositol (GPI) and GPI-anchored proteins. Thus, compound **A1** represents a promising lead compound to combat drug-resistant candidiasis.

## GRAPHICAL ABSTRACT

**Figure:**
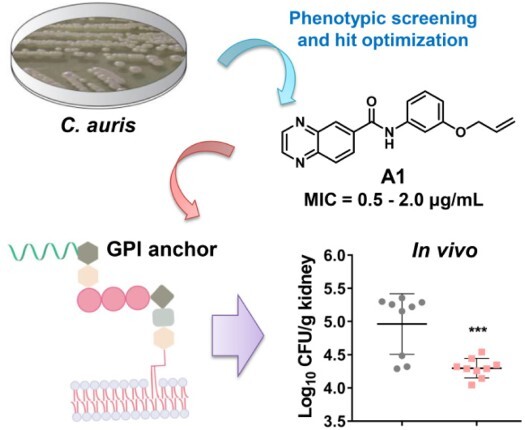

## Introduction

The emergence of invasive fungal infections (IFIs) has become a global threat to human health in recent decades [1,2]. Fungal diseases caused by drug-resistant pathogens are estimated to kill nearly 2 million people and infect billions of immunocompromised populations each year, including patients undergoing cancer chemotherapy and organ transplant and infected with human immunodeficiency virus (HIV) [3,4]. Recently, IFIs have been reported to be prevalent in patients with coronavirus disease 2019 (COVID-19) and associated with high mortality [5,6]. The main opportunistic fungal pathogens include *Candida*, *Cryptococcus* and *Aspergillus* species, which account for nearly 90% of deaths in IFIs [2,7]. The development of drug-resistant fungal species further contributed to therapeutic failure in the clinic [8].

*Candida* species are the major cause of IFIs in severely immunocompromised populations [9]. *Candida albicans* (*C. albicans*), featured with ever-increasing pathogenicity and severe drug resistance [10,11], caused life-threatening *Candida* bloodstream infections [2,12]. Recently, *Candida auris* (*C. auris*) has emerged as a serious threat of hospitalized invasive infections and caused worldwide concern due to its severe drug resistance, high mortality and remarkable transmissibility [13,14]. *C. auris* belongs to the clade of *Candida haemulonii* [15]. and the body infection sites of *C. auris* colonization mainly include skins, mucosa and gastrointestinal tract, which are similar to those observed for other common *Candida* species [16]. However, *C. auris* has a stronger colonization ability to persist on host skins than other *Candida* species, which causes nosocomial transmission and infections among immunocompromised individuals [17,18]. This emerging pathogen has rapidly spread to over 40 countries across several continents, further demonstrating the detrimental impact of drug-resistant fungal infections.

Clinically, only a few types of antifungal agents (i.e. azoles, polyenes, echinocandins and pyrimidines) are currently applicable to addressing these life-threatening fungal pathogens [19,20]. However, most *C. auris* strains were reported to be multi-drug resistant [18]. *C. auris* is the only *Candida* species that was identified to be resistant to all four classes of antifungal drugs [16]. Despite the increasing clinical requirements, the development of new antifungals is sparse in recent years due to the eukaryotic properties of fungal cells and poor permeability of compounds to the cell wall. Currently, most active compounds against *C. auris* were identified by re-evaluations of known antifungal agents (compounds) or reposition of existing drugs [21]. Unfortunately, the efficacy of these therapies against *C. auris* remains too limited to enter clinical practice [22–26]. The lack of effective agents and prevalence of resistance highlight the urgent need for the discovery of novel antifungals against *C. auris*, particularly those with novel chemical scaffolds, good therapeutic effects and distinct mechanisms of action.

**Figure 1.**
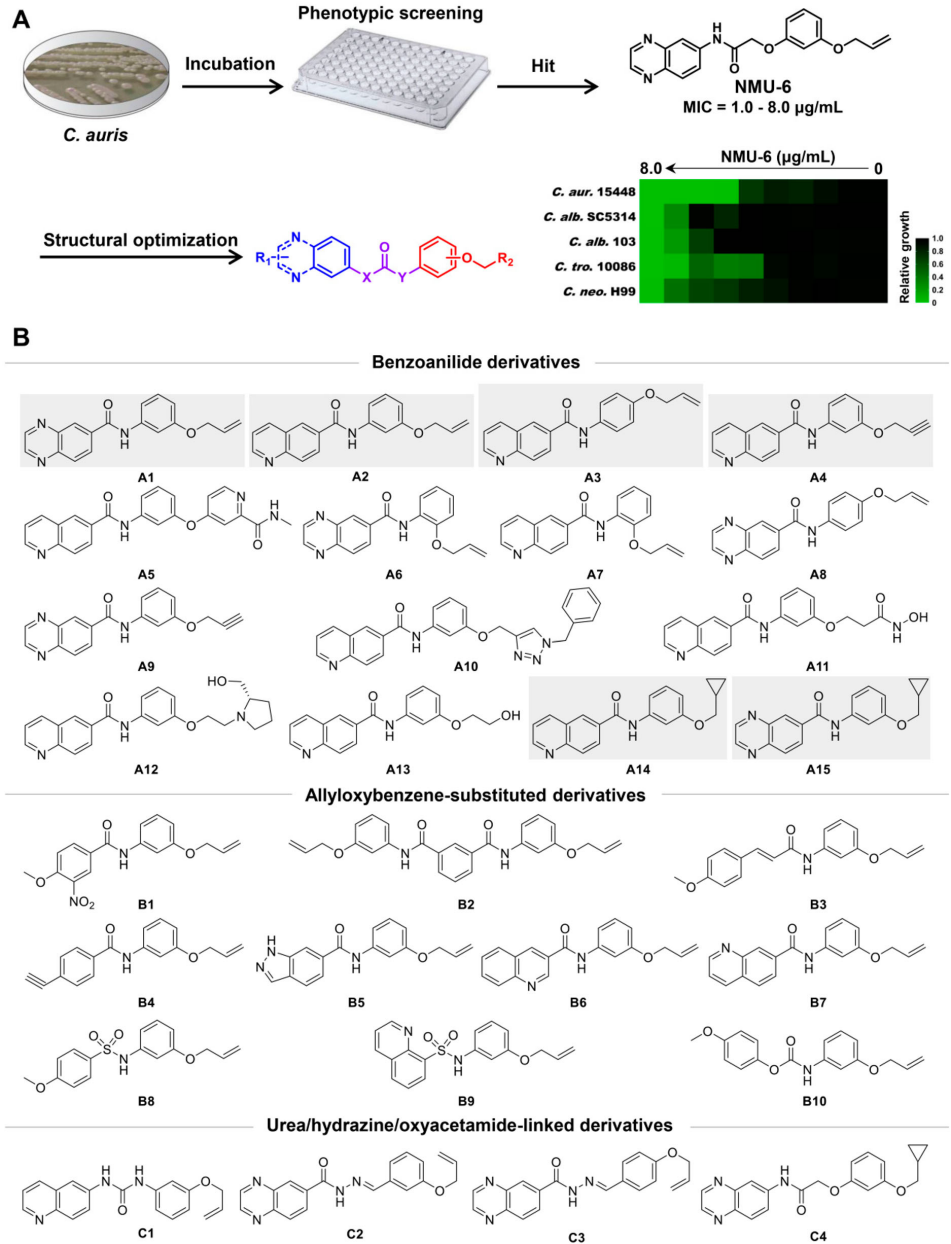
Discovery of benzoanilide lead compounds against *C. auris*. (a) Protocol for phenotypic screen and molecule design based on the hit compound **NMU-6**. Relative growth of fungal cells was displayed by heatmap. (b) Chemical structures of the designed target compounds. The shaded regions indicate compounds that showed potent antifungal activity.

Phenotypic screen of large-scale chemical libraries is an efficient strategy for the discovery of novel antifungal lead compounds [27–29]. Particularly, identification of compounds with new chemical scaffold and antifungal mechanism was highly desirable to overcome drug resistance. Previously, our group identified highly potent carboline antifungal agents by phenotypic screen and follow-on structural optimizations [27,30], providing a basis for the discovery of new antifungal chemotypes. Herein, we performed an antifungal screen of an in-house library against azole-resistant *C. auris*. **NMU-6** was identified as a hit compound that exhibited an MIC value of 1.0 μg/mL against *C. auris* (strain number: 15448), representing a good starting point for the discovery of novel antifungals against *C. auris*. After rapid establishment of structure–activity relationships (SARs), a series of novel benzoanilide derivatives with improved antifungal potency were successfully identified (Figure 1(b)). In particular, compound **A1** exhibited excellent *in vitro* and *in vivo* antifungal activity in treating drug-resistant *Candida* species including *C. auris* and *C. albicans*. Further antifungal mechanism characterization of compound **A1** revealed that it acted by inhibiting the virulence factors and biosynthesis of fungal cell wall through the inhibition of glycosylphosphatidylinositol (GPI) and GPI-anchored proteins.

## Materials and methods

### Strains and culture

Strains were routinely incubated in YPD (1% yeast extract, 2% peptone and 2% dextrose) at 30°C in a shaking incubator. *C. albicans* SC5314 and *C. neoformans* H99 were experimental standard strains, and clinically isolated *C. auris* 0029, *C. auris* 0030, *C. auris* 15448, *C. albicans* 103, *C. glabrata* 7669, *C. lusitaniae* 9150, *C. parapsilosis* 20090, and *C. tropicalis* 10086 were provided by Shanghai Changzheng Hospital. HUVEC cells were derived from Shanghai Cell Bank, Chinese Academy of Sciences.

### Compounds

All compounds were dissolved in DMSO at 2 mg/mL as stock solutions. The chemical synthesis of target compounds was depicted in **Schemes S1–S4**.

### In vitro antifungal activity

The fungal cells cultured in YEPD to the exponential growth phase were collected, washed three times with PBS, and diluted with RPMI 1640 to a fungal suspension of 1.0 × 10^3^ CFU/mL. The fungal suspension prepared above was added to columns 1–11 in 96-well plates, and the compounds to be tested were added to the first column and diluted 1/2 from 1 column to 10 columns. In the 96-well plates, only the fungal suspension was added in the 11th column as a positive control, and only RPMI 1640 was added in the 12th column as a negative control, and each compound concentration was made three wells. The 96-well plates were subsequently incubated at 35°C for 48 h (*C. neoformans* H99 needed to be incubated for 72 h). The absorbance A of each well at a wavelength of 630 nm was measured using a microplate reader. According to the formula: inhibition per well (%) = (A _positive control well_-A _compound well_) / (A _positive control well_-A _negative control well_) × 100%, the inhibition ratio corresponding to each compound concentration was calculated, and the MIC value of the compound was the minimum compound concentration with an inhibition rate higher than 80%.

### Time-growth curve assay

Fungal cells of *C. albicans* 103 and *C. auris* 0029 were incubated to the exponential growth phase and collected, washed three times with PBS, then resuspended in RPMI and counted. The above fungal suspension was half diluted 10 times and the corresponding absorbance values were recorded, after which a standard curve was made. After counting, RPMI 1640 medium was used to dilute the fungal suspension to 1.0 × 10^6^ CFU/mL. The above fungal suspension (5 mL) was added to a sterile centrifuge tube, and different concentrations of compounds **A1**, **A2** and FLC were added to each tube and incubated at 35°C for 48 h. Fungal cultures (3 × 100 μL) of each drug concentration group at 0, 3, 12, 24, 36 and 48 h was added to the 96-well plates, and OD_630_ values were measured. OD_630_ values were converted into fungal counts by the standard curve to draw a time-growth curve.

### Biofilm formation assay

Fungal cells of *C. albicans* 103 and *C. auris* 0029 were incubated to the exponential growth phase and collected, washed three times with PBS, then RPMI 1640 medium was used to dilute the fungal suspension to 1.0 × 10^6^ CFU/mL. The fungal cell suspension was added to 96-well plates and incubated at 37°C for 1.5 h (24 h for mature biofilm formation). The RPMI 1640 medium in 96-well plates was discarded, and the fungal cells without adhesion were washed off with PBS. Compounds at different concentrations were added and incubated at 37°C for 24 h with FLC as a positive control. Finally, the culture medium was discarded and XTT/menadione solution was added, and the OD_490_ value of each well was measured after avoiding light for 3 h to calculate the biofilm formation rate.

### Hypha growth assay

Fungal cells of *C. albicans* 103 in the exponential growth phase were collected, washed three times with PBS, then spider was used to dilute the fungal suspension to 1.0 × 10^6^ CFU/mL. Fungal suspensions (1.5 mL) were added to 12-well plates and distributed evenly at the bottom of the plates, then compounds at different concentrations were add and FLC was used as a positive control, and finally plates were placed at 37°C for 3 h. Morphological differences between drug-treated and no-drug-treated groups were recorded using an inverted microscope.

### In vivo antifungal potency

The experimental animal study was approved by the Committee on Ethics of Medicine of the Second Military Medical University. Fungal cells in the exponential growth phase were collected, washed three times with PBS, then physiological saline was used to dilute the fungal suspension to 3.0 × 10^5^ CFU/mL. ICR female mice (18–20 g, Shanghai Experimental Animal Center, Certificate SCXK-2007-0005) were injected fungal suspension (*C. albicans* 103 or *C. auris* 0029, 0.2 mL) via the tail vein to create a mouse model of invasive fungal infection. Mice were dosed after 2 h, mice in different compound groups were treated with intraperitoneal injection or intragastric administration, once a day at fixed time, and remained dose for 5 days. At the end of administration, the mice were euthanized on day 6, the kidneys of mice were aseptically enucleated for homogenization, and the appropriate concentrations of the homogenates were inoculated onto SDA plates and incubated for 48 h. Finally, the number of colonies generated on the SDA plate was counted, and the amount of fungal colonies in the kidney tissue was calculated.

### TEM observation assay

*C. auris* 0029 and *C. albicans* 103 in exponential growth phase were washed with PBS and diluted by RPMI 1640 medium to 2.0 × 10^6^ CFU/mL. DMSO, FLC, and compound A1 were added to the fungal suspension and incubated at 30°C for 8 h. Strains were collected, washed, resuspended in fixative solution containing 4% paraformaldehyde and settled overnight at 4°C. Samples were washed with PBS and fixed in 1% phosphotungstic acid for 90 min, dehydrated and embedded in EPON-812. After double staining using uranium and lead, ultrathin sections were prepared and observed under a transmission electron microscope (Hitachi H-800, Japan).

### Mannan fluorescence assay

*C. auris* 0029 and *C. albicans* 103 in the exponential growth phase were collected, and fungal suspension (200 μL) was added to YEPD solution (20 mL), and compound **A1** (4.0 μg/mL) was added to co-culture for 4 h. The supernatant was removed by centrifugation, washed three times with PBS, and stained with ConA (20 μg/mL) for 1 h. The stain was removed by centrifugation, washed three times with physiological saline, and resuspended in physiological saline (400 μL). The fungal suspension was thoroughly mixed before taking photographs and fungal suspension (6.0 μL) each time was taken to a sticky glass slide for fluorescent photographs.

### β-Glucan fluorescence assay

Fungal cells of *C. auris* 0029 and *C. albicans* 103 in the exponential growth phase were collected, and fungal suspension (200 μL) was added to YEPD solution (20 mL), and compound **A1** (4.0 μg/mL) was added to co-culture for 4 h. The supernatant was removed by low-speed centrifugation, washed three times with PBS and blocked with 2% BSA/PBS for 30 min. The blocking solution was removed by centrifugation, and freshly prepared β-glucan antibody incubation solution (200 μL) was added to incubate at 37°C for 1 h. β-Glucan antibody incubation solution was removed by centrifugation, and goat anti-mouse stain (200 μL) was added to incubate at 37°C for 40 min. The stain was removed by centrifugation, washed three times with physiological saline, and resuspended in physiological saline (400 μL), the fungal suspension was thoroughly mixed before taking photographs and fungal suspension (6.0 μL) each time was taken to a sticky glass slide for fluorescent photographs.

### TNF-α assay by co-culture of macrophages and fungi

The resuscitated macrophages RAW264.7 were incubated in DMEM complete medium for 24 h. The cells were washed with cold PBS and centrifuged after adhered completely, and resuspended in DMEM medium. Macrophage culture solution (1.0 × 10^6^ CFU/mL) was added to 12-well plates, the drug-treated fungal cells that need to be inactivated by UV before adding *C. auris* 0029 and *C. albicans* 103 (Fungal cells/macrophages = 1/2), and finally 12-well plates were placed at 37°C for 2 h. The medium in the 12-well plates was placed in EP tubes, and the cell pellet was removed by high-speed centrifugation, and the supernatant was dispensed into an EP tube for testing. The TNF-α in the medium was operated strictly according to the kit, the value was recorded, and statistical analysis was performed.

### GPI anchoring level assay

Fungal cells of *C. auris* 0029 and *C. albicans* 103 in the exponential growth phase were collected, washed three times with PBS, and then PBS was used to dilute the OD_600_ value to 0.20. Fungal cells were incubated with FLAER (20 nM) for 30 min on ice, washed three times with physiological saline, and resuspended in physiological saline (400 μL), and fungal suspension (6.0 μL) was taken to the sticky glass slide for fluorescent photographs.

### Real time RT-PCR assay

Fungal cells of *C. albicans* 103 frozen at −80°C were inoculated and cultured in YEPD medium (1.0 mL) for 24 h, after which fungal suspension (100 μL) was added to YEPD (30 mL) and different drug concentrations for co-cultivation for 24 h, and drug-free group was set as a control. After co-cultivation, the supernatant was removed by centrifugation, washed three times with PBS, and dissolved in PBS (1 mL). Add the above-prepared fungal suspension to the ceramic mortar and slowly add liquid nitrogen to grind well until a uniformly sized fungal mass is visible in the mortar to stop grinding, next add the ground fungal mass to a sterile EP tube (1.5 mL) with a sterile iron spoon. Subsequently, the total fungal RNA was extracted with reference to the RNeasy Plant Mini kit (QIAGEN), and the RNA concentration was determined with a microspectrophotometer. Using the extracted total RNA as a template, the reverse transcription reaction system was prepared by mixing reverse transcriptase (10 μL) + RNA (2500 ng) + appropriate sterilized water, and the cDNA of each group was obtained under reverse transcription conditions. The cDNA obtained was used to amplify the genes according to qPCR reaction conditions using actin as internal reference to calculate the C_T_ values for each group. The relative expression of genes was calculated by formula 2^(−ΔΔC^_T_^)^. The expression multiple of each gene in different groups was calculated, and the gene expression level of each group was compared by Student's *t*-test. The gene primer sequences used in the experiment were depicted in Table S4.

### Statistical analysis

All experiments were performed in triplicates unless specified otherwise. GraphPad Prism Software 7.04 was used for all statistical analyses. Results were presented as mean ± SD. Specific statistical tests were indicated in the figure legends. *P < 0.05 was considered significant.

## Results

### Discovery of an anti-C. auris hit by phenotypic screen

To identify novel antifungal compounds against *C. auris*, we screened an in-house library by microdilution method in 96-well culture plates (Figure 1(a)). The antifungal activities of target compounds were expressed as the minimum inhibitory concentration (MIC) that achieved 80% inhibition against *C. auris*. We prioritized compound **NMU-6** as a primary hit due to its strong antifungal activity against azole-resistant *C. auris* 15448 (MIC = 1.0 μg/mL). The antifungal spectrum of compound **NMU-6** was further assayed against *C. albicans* SC5314 (azole-sensitive strain), *C. albicans* 103 (azole-resistant strain), *Candida tropicalis* (*C. tropicalis*) 10086 and *Cryptococcus neoformans* (*C. neoformans*) H99. The results showed that compound **NMU-6** was also active against these species (MIC range: 1.0–8.0 μg/mL, Figure 1(a)).

Considering the potent anti-*C. auris* activity and broad spectrum of compound **NMU-6**, further structural optimization and SAR analysis were performed. The chemical structure of compound **NMU-6** is featured as a quinoxalinamide scaffold, an allyloxybenzene terminal group and an oxyacetamide linker. Then a series of new derivatives were designed and synthesized focusing on the variation of benzanilide scaffold, linker and terminal phenyl substitutions (Figure 1(b)).

### SAR explorations identified benzoanilide lead compounds with improved antifungal activities

**Figure 2.**
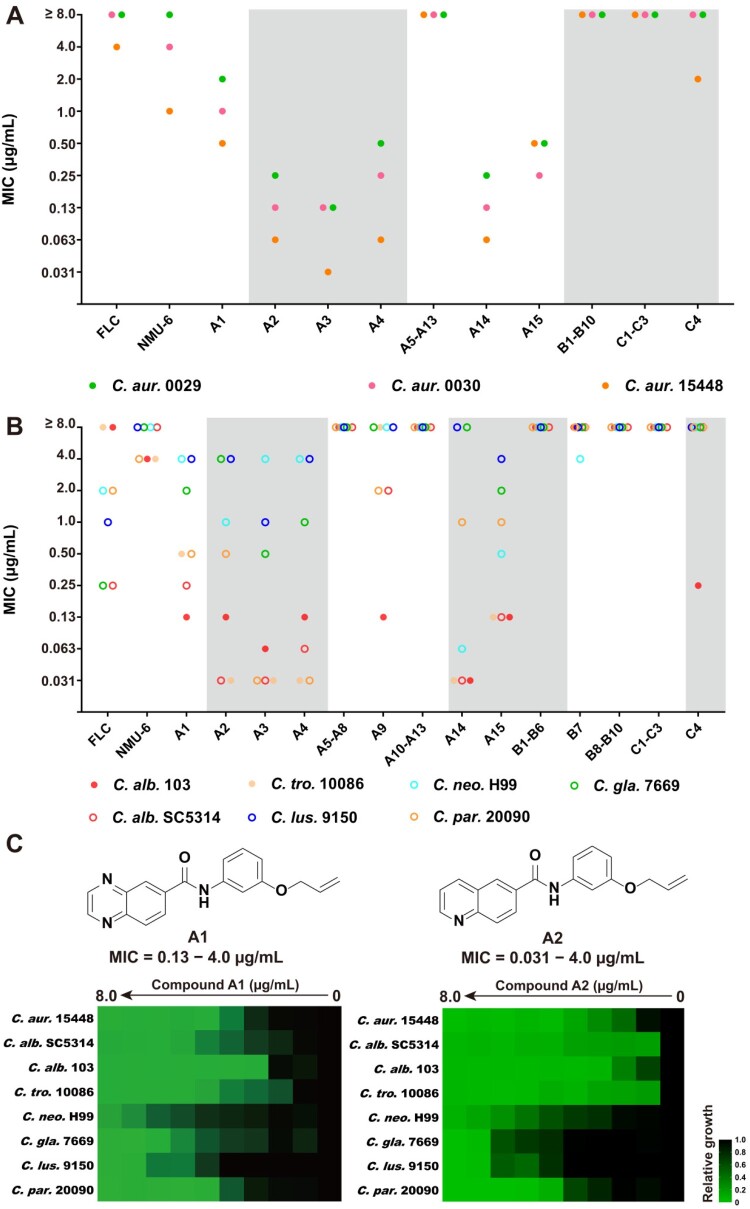
Selection of compounds **A1** and **A2** as potent antifungal lead compounds. (a) The antifungal activities of compounds against *C. auris*. (b) The antifungal activities of compounds against *C. albicans*, *C. tropicalis* and *C. neoformans*, filled and empty circles indicate FLC-resistant and FLC-sensitive strains, respectively. (c) The antifungal potency of compounds **A1** and **A2**. Relative growth of fungal cells was displayed by heatmap. Abbreviations: *C. alb.*, *Candida albicans*; *C. tro.*, *Candida tropicalis*; *C. neo.*, *Cryptococcus neoformans*; *C. aur.*, *Candida auris*; *C. gla*., *Candida glabrata*; *C. lus*., *Candida lusitaniae*; *C. par*., *Candida parapsilosis*.

In order to elucidate the SARs, all the target compounds were assayed against three *C. auris* isolates. Compounds **A1–A15** were featured as the benzoanilide scaffold, in which compounds with the quinolone group and the meta/para-substituted side-chain (compounds **A2–A4**, and **A14**) exhibited better inhibitory activity than compounds with a large or ortho-substituted side-chain (compounds **A5** and **A7**). In contrast, quinoxaline-substituted derivatives with an ortho/para-substituted side-chain were totally inactive (compounds **A6** and **A8**), compounds **A1**, **A9** and **A15** with the meta-substituted side-chain exhibited obviously improved antifungal activity. However, meta-substituted derivatives with a long chain lost antifungal activity (compounds **A10–A13**). Replacement of the benzoanilide scaffold by substituted phenyl groups or other heterocycles (compounds **B1–B10**) led to the loss of the inhibitory activity against all the tested *C. auris* isolates. Compounds **C1–C3** with the urea or hydrazine linker were inactive, while the introduction of oxyacetamide linker (compound **C4**) resulted in improved antifungal activity. Compounds **A1–A4** and **A14–A15** exhibited potent activity against azoles-resistant *C. auris* (Figure 2(a)), which were more effective than fluconazole (FLC). In particular, compounds **A1** and **A2** were highly active against *C. auris* isolates (MIC range: 0.06–2.0 μg/mL).

To investigate the broad-spectrum efficacy of the target compounds, the antifungal assay was further expanded to *C. albicans*, *C. tropicalis*, *C. glabrata*, *C. lusitaniae*, *C. parapsilosis*, and *C. neoformans* (Figure 2(b) and Table S2). The SARs against these tested fungal pathogens were almost consistent with those for *C. auris*. Compounds **B1–B6** and **B8–B10** were inactive against all the tested strains. 3-Quinoline derivative **B7** showed moderate antifungal activities against *C. neoformans* (MIC = 4.0 μg/mL). Compounds **A1–A4** and **A14–A15** exhibited potent activity against *C. albicans*, *C. tropicalis*, and *C. parapsilosis* species (MIC range: 0.031–1.0 μg/mL) including drug-resistant isolates. Benzoanilide derivatives **A1** and **A2** showed excellent antifungal activity against the tested fungal pathogens, which were superior to FLC and lead compound **NMU-6** (Figure 2(c)). The cytotoxicity of these benzoanilide derivatives were also assessed (Table S3). The results revealed that compounds **A1** and **A2** showed relatively low toxicity on human normal cell line HUVEC (IC_50_ > 29 μg/mL). Therefore, compounds **A1** and **A2** were selected for further evaluations.

### Compounds A1 and A2 effectively inhibited biofilm formation of C. auris

**Figure 3.**
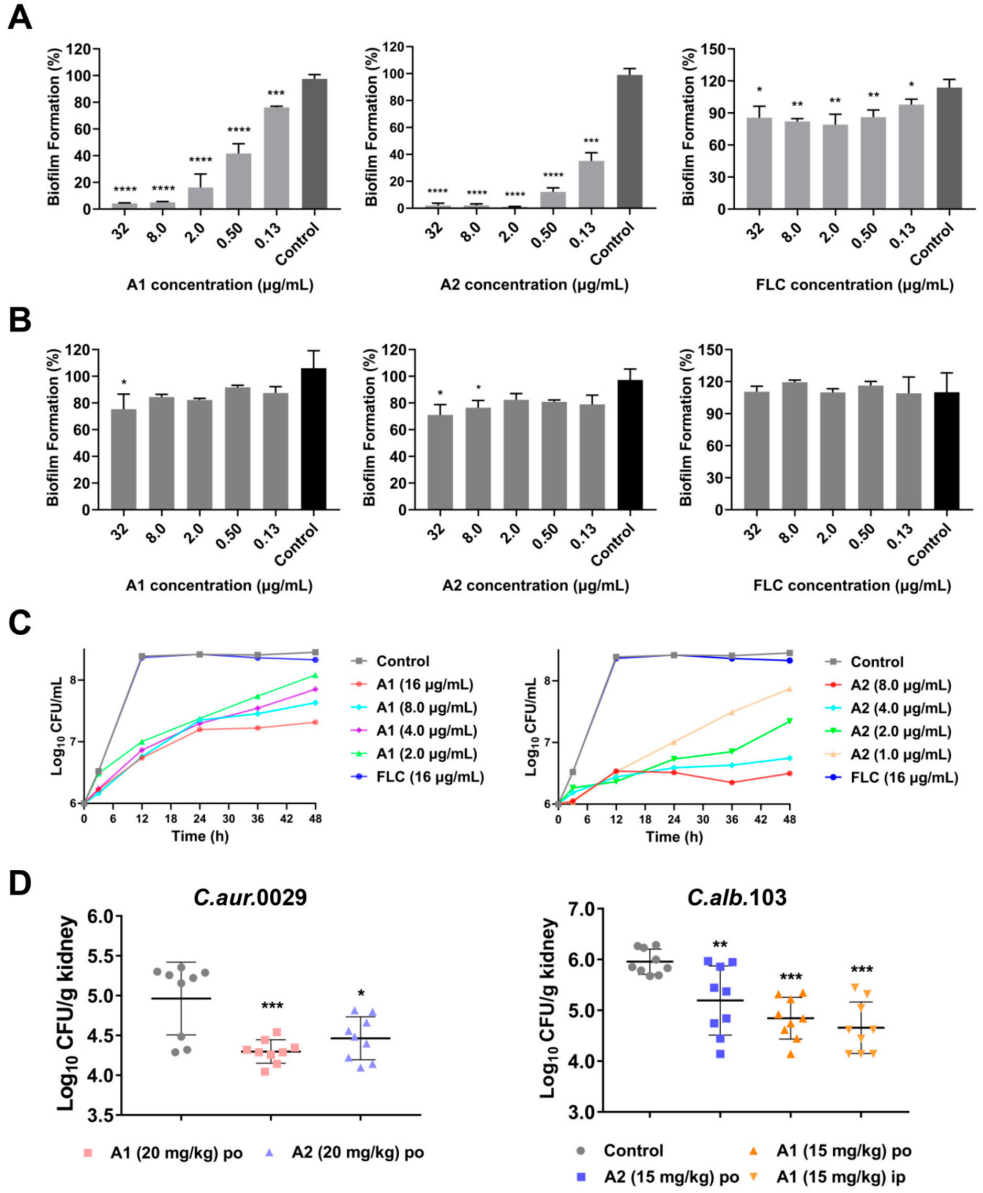
*In vitro* and *in vivo* antifungal activities of compounds **A1** and **A2** against *C. auris*. (a) Inhibition of compounds **A1**, **A2** and FLC on *C. auris* 0029 biofilm formation and (b) mature biofilm. (c) Time-growth curve of *C. auris* 0029 treated by compound **A1**, **A2** and FLC. (d) *In vivo* antifungal efficacy of compounds **A1** and **A2** in IFIs mice model, fungal burden of kidneys in mice infected by *C. auris* 0029 or *C. albicans* 103. *P < 0.05, **P < 0.01, ***P < 0.001, and ****P < 0.0001, vs. the control group, determined by one-way ANOVA.

Similar to other *Candida* species, *C. auris* is able to form biofilms that are related to severe virulence and resistance [31]. To confirm whether compounds **A1** and **A2** possessed anti-biofilm properties, a series of dose–response assays on fungal biofilms were firstly performed according to the well-established protocols [32]. As shown in Figure 3(a), FLC exhibited weak inhibition on the biofilm formation of *C. auris* 0029 (∼18% inhibition rate at 32 μg/mL). In contrast, compounds **A1** and **A2** could significantly suppress the biofilm formation at the concentration of 0.13 μg/mL with an inhibition rate of 24% and 65%, respectively. With the increase of the concentrations, the inhibition rate was slightly improved and finally ∼95% inhibition was achieved at the concentration of 32 μg/mL. For the mature biofilm, compounds **A1** and **A2** had modest inhibitory effect under the high concentrations, whereas FLC was totally ineffective (Figure 3(b)).

### Compounds A1 and A2 showed potent inhibitory activity against drug-resistant C. auris and C. albicans

**Figure 4.**
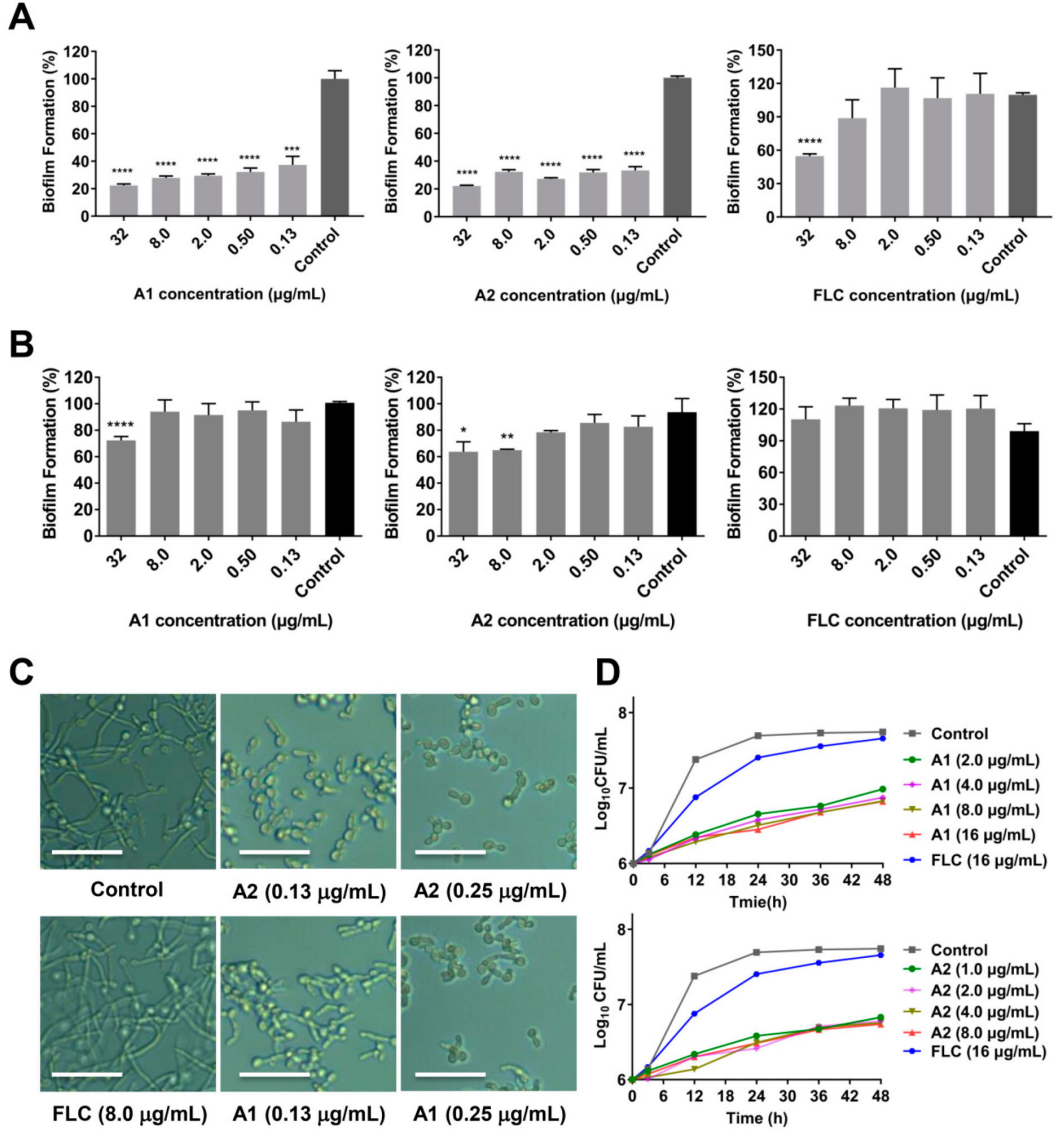
Anti-virulence effects of compounds **A1** and **A2** against azole-resistant *C. albicans* 103. (a) Inhibition of biofilm formation; (b) Inhibition of mature biofilm; (c) Inhibition of hypha formation, scale bar represents 50 µm. (d) Time-growth curve of *C. albicans* 103 treated by compounds **A1**, **A2** and FLC. *P < 0.05, **P < 0.01, ***P < 0.001, and ****P < 0.0001, vs. the control group, determined by one-way ANOVA.

To further evaluate the antifungal profile of compounds **A1** and **A2** against *C. auris* 0029 and *C. albicans* 103, time-growth curves assay was performed. As shown in Figure 3(b) and Figure 4(d), FLC did not show obvious inhibition of *C. auris* 0029 and *C. albicans* 103 at a high concentration of 16 μg/mL. In contrast, compounds **A1** (1.0 μg/mL) and **A2** (2.0 μg/mL) exhibited excellent fungistatic activity rather than fungicidal effects. Both of them could continuously inhibit the proliferation of fungal cells within 48 h.

### Compounds A1 and A2 inhibited the virulence factors of drug-resistant C. albicans

On the basis of the potency of compounds **A1** and **A2** against *C. auris* isolates, their antifungal effects were further evaluated against drug resistant *C. albicans*. Filamentation and biofilm formation have been recognized as critical virulence factors of *C. albicans*, which are closely related to the pathogenicity and drug resistance [11]. To confirm whether compounds **A1** and **A2** possessed anti-virulence activity against *C. albicans*, hyphae and biofilm formation assays were also performed against drug-resistant *C. albicans* 103 isolates. Similar to anti-biofilm properties against *C. auris* 0029, compounds **A1** and **A2** could significantly suppress the biofilm formation of *C. albicans* 103 at the concentration of 0.13 μg/mL (67% and 63% inhibition rate, respectively), and finally achieved ∼80% inhibition at the concentration of 32 μg/mL, whereas FLC only exhibited weak inhibition at the concentration of 32 μg/mL with an inhibition rate of ∼50% (Figure 4(a)). For the mature biofilm, compounds **A1** and **A2** had modest inhibitory effects under the high concentrations, whereas FLC was totally ineffective (Figure 4(b)). Compounds **A1** and **A2** also effectively inhibited the yeast-to-hypha transition process of *C. albicans*. As depicted in Figure 4(c), almost complete inhibition of hypha formation was observed at the concentration as low as 0.25 μg/mL for compounds **A1** and **A2**, whereas FLC was inactive at 8.0 μg/mL.

### Compounds A1 exerted potent in vivo antifungal activity against C. auris and C. albicans infections

The *in vitro* assays revealed that compounds **A1** and **A2** exhibited excellent antifungal and anti-virulence activities against azoles-resistant *C. auris* and *C. albicans* isolates. Two candidiasis mice models were established via tail vein injection of fungal cells to evaluate the *in vivo* antifungal potency of compounds **A1** and **A2**. In the azole-resistant *C. auris* 0029 infection model (Figure 3(d)), oral (PO) administration of compound **A1** at the dose of 20 mg/kg significantly reduced the fungal burden in the kidney of mice (*P* < 0.001), which was more effective than the **A2**-treated group (*P* < 0.05). In the *C. albicans* 103 infection model (Figure 3(d)), treatment with compounds **A1** and **A2** (15 mg/Kg) by oral administration also significantly alleviated the fungal burden in the kidney (*P* < 0.01), of which the **A1**-treated group had a better efficacy (*P* < 0.001). In addition, the intraperitoneal (IP) administration of compound **A1** was evaluated, in which similar antifungal potency was observed (*P* < 0.001). The results highlighted the potential of compound **A1** for the treatment of drug-resistant candidiasis.

### Compound A1 destroyed mannan coats and unmasked β-glucan of fungal cell walls

**Figure 5.**
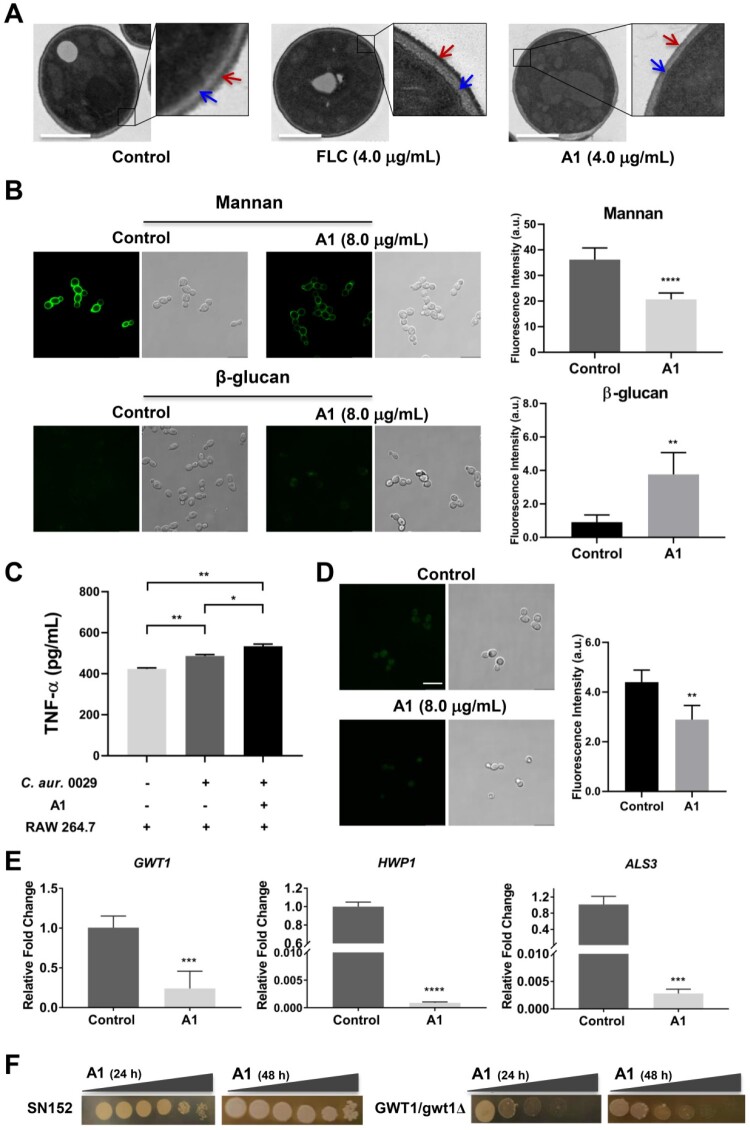
Mechanism-of-action of compound **A1**. (a) Transmission electron images of drug-resistant *C. auris* 0029 after incubating with FLC and compound **A1**. The red and blue arrows indicate the mannan coats and β-glucan layer, respectively. Scale bar represents 1 µm. (b) Fluorescence intensity assay of mannan and β-glucan in *C. auris* 0029 cell walls after treatment with compound **A1**. Scale bar represents 10 µm. **P < 0.01, and ****P < 0.0001, vs. the control group, determined by two-tailed unpaired Student’s *t*-test. (c) The TNF-α concentration analysis by co-culture of macrophages RAW264.7 and *C. auris* 0029 treated by compound **A1** (8.0 μg/mL). *P < 0.05, and **P < 0.01, determined by one-way ANOVA. (d) FLAER staining of GPI-anchored proteins in fungal cells. Scale bar represents 10 µm. **P < 0.01, vs. the control group, determined by two-tailed unpaired Student’s *t*-test. (e) The regulatory effects of compound **A1** on *GWT1*, *HWP1*, and *ALS3* genes of *C. albicans* 103 through real-time RT-PCR analysis. ***P < 0.001, and ****P < 0.0001, vs. the control group, determined by two-tailed unpaired Student’s *t*-test. (f) Effects of GWT1 knockdown on drug sensitivities. Plates contained increasing concentrations of compound **A1** to 0.25 μg/mL.

Compound **A1** showed potent antifungal efficacy both *in vitro* and *in vivo*, whereas its mechanism-of-action was still unclear. Microscopic observation assay of fungal morphology is an effective approach to investigate antifungal mechanism, which allows visual analysis of the injury to fungal cells [27,33,34]. The drug-resistant mechanisms of *C. auris* are similar to those observed in other *Candida* species [35]. Thus, azole-resistant *C. auris* 0029 and *C. albicans* 103 were used to as model strains to investigate the antifungal mechanism. The morphological changes of *C. auris* 0029 cells treated by compound **A1** and FLC were observed by transmission electron microscopy (TEM). As shown in Figure 5(a), normal *C. auris* 0029 cells have a complete fungal cell wall with dense mannan coats and clear outline of β-glucan. No obvious damage of cell wall was observed in *C. auris* 0029 cells treated with 4.0 μg/mL of FLC, in which the β-glucan was normally masked by mannan coats. Interestingly, the mannan coats of *C. auris* 0029 cells became thinner and even disappeared after the incubation with compound **A1** (4.0 μg/mL), resulting in the exposure of inner β-glucan (Figure 5(a)). The fluorescence intensity assay was performed to further investigate the effects of compound **A1** on the contents of mannan and β-glucan (Figure 5(b)), which was well consistent with the results from the microscopic observation. After the treatment with compound **A1** (8.0 μg/mL) for 4 h, the fluorescence intensity of mannan layer in *C. auris* 0029 cells decreased significantly (*P* < 0.0001), while β-glucan was significantly increased by 4.1-fold (*P* < 0.01).

### Compound A1 enhanced immune recognition

The exposure of β-glucan in the fungal cell walls was a kind of pro-inflammatory stimulus, leading to the secretion of the immune factor TNF-α and enhanced recognition of fungi by immune cells of infected hosts [36]. Thus, the TNF-α concentration was analyzed (Figure 5(c)), and the co-culture of *C. auris* 0029 cells and mouse macrophages RAW264.7 was able to increase the TNF-α level (*P* < 0.01). Notably, the **A1**-treated *C. auris* 0029 cells (8.0 μg/mL) could significantly enhance the secretion of TNF-α (*P* < 0.05) compared to control-treated fungi. Thus, in addition to directly killing fungal cells by destroying the cell walls, compound **A1** also enhanced host immune response through the β-glucan exposure, thereby exerting *in vivo* antifungal efficacy.

### Compound A1 acted by inhibiting the biosynthesis of GPIs and GPI-anchored proteins

The microscopic observation assay and verification of β-glucan exposure revealed that the cell walls of *C. auris* 0029 were damaged by treatment with compound **A1**. GPIs and GPI-anchored proteins play a vital role in diverse fungal biological processes, including adherence, virulence, and cell wall biogenesis [37–40]. Recently, an increasing number of GPI inhibitors, mainly including Gwt1 inhibitors and Mcd4 inhibitors, have been identified to possess excellent antifungal efficacy [41–43]. To validate whether compound **A1** acted on the GPI-related pathway, a series of assays were carried out. FLAER, an aerolysin tagged with Alexa 488 fluorophores, could selectively bind to GPI-anchored proteins in fungal cells [40]. As shown in Figure 5(d), upon incubating the *C. auris* 0029 cells with FLAER, the bright green clusters of GPI anchor on the fungal cell walls were observed. Interestingly, in comparison with the control group, the fluorescence intensity of fungal cells was significantly decreased (**P < 0.01) after the treatment of compound **A1** (8.0 μg/mL) for 1 h.

Moreover, the above antifungal mechanism studies were also performed on FLC-resistant *C. albicans* 103 (Figure S1), and the results were highly consistent with those of the *C. auris* 0029, further confirming the mechanism of action of compound **A1**. Thus, the real-time RT-PCR assay was performed on *C. albicans* 103 to investigate the expression levels of GPI-related genes, due to the unavailability of GPI-related gene primers of *C. auris*. Considering the anti-virulence effects of compound **A1**, the expression of adhesion-associated *ALS3* and biofilm-associated *HWP1* genes, and genes involved in biosynthesis of GPI-anchored proteins was evaluated. As depicted in Figure 5(e), the treatment of compound **A1** (0.50 μg/mL) significantly down-regulated the expression of *GWT1* (*P* < 0.001), *HWP1* (*P* < 0.0001), and *ALS3* (*P* < 0.001) genes. The Gwt1 inhibitory potency of compound **A1** was verified by GWT1 knockdown strains (Figure 5(f)). The reference strain *C. albicans* SN152 was slightly inhibited by compound **A1** at the highest concentration of 0.25 μg/mL. Notably, compound **A1** exhibited obviously increased sensitivity to GWT1 knockdown *C. albicans*. The above results indicated that compound **A1** could effectively inhibit the biosynthesis of GPIs and GPI-anchored proteins.

## Discussion

In recent years, with the emergence and widespread of multidrug-resistant *C. auris*, there is an urgent need to develop effective therapeutics [13,14]. Currently, the understanding of epidemiology, virulence factors and drug resistance of *C. auris* is rather limited [16,44]. It is highly challenging to identify promising targets and potent anti-*C. auris* agents [45]. Screening of known antifungal agents or clinical antifungal candidates has yielded active compounds [22–26]. However, their potential applications have not been validated clinically. Alternatively, drug repurposing of marketed drugs also identified lead compounds or synergistic drug combinations [46–48], whereas they could hardly be used directly because of limited potency and selectivity. In this study, we screened an in-house compound library, which were previously constructed with structure diversity and drug-likeness [27,49]. **NMU-6** was identified as a hit that exhibited potent anti-*C. auris* activity (MIC = 1.0 μg/mL). It also showed broad-spectrum antifungal activities against *C. albicans*, *C. tropicalis* and *C. neoformans* H99 (MIC range: 1.0–8.0 μg/mL).

From a medicinal chemist point of view, **NMU-6** possesses a new chemical scaffold that was distinct from known anti-*C. auris* compounds [21]. Notably, most screening hits active against *C. auris* have not been optimized, whose SARs and antifungal mechanism are still unknown [50]. Thus, further development of these active compounds was hampered. Herein an integrated approach was used to establish SARs, improve the potency and clarify the antifungal mechanism. The SARs of **NMU-6** were investigated by the variation of heterocyclic cap group, linker and terminal phenyl substitutions. Benzopyrazine (or quinoline) cap and amide linker seemed to be essential for the antifungal activity. The terminal substitution could be tolerated by vinyl, ethynyl cyclopropyl groups. It should be noted that only a limited set compounds were synthesized and detailed SAR exploration is necessary for structural optimization.

SAR analysis and structural optimization successfully led to the discovery of two highly active derivatives. Compared with FLC and **NMU-6**, benzoanilide derivatives **A1** and **A2** exhibited improved antifungal activity against *C. auris* isolates and *Candida* species with the MIC range of 0.06–2.0 μg/mL and 0.031–4.0 μg/mL, respectively. Of note, our data have demonstrated that these new benzoanilide compounds also displayed *in vivo* antifungal potency in azoles-resistant *C. auris* and *C. albicans* mice models. Although compound **A2** generally had lower MIC values than compound **A1**, compound **A1** showed better *in vivo* antifungal antifungal potency than compound **A2**. A possible reason is that compound **A1** might have more favourable pharmacokinetic profiles, which remained to be validated experimentally. Compound **A1** was orally active and showed potent *in vivo* antifungal efficacy both in azole-resistant *C. auris* 0029 and *C. albicans* 103 infection models, highlighting the potential of this new chemical scaffold for the treatment of drug-resistant candidiasis.

Fungal cells and human cells are both eukaryotic, which shares high similarity. Thus, selectivity and cytotoxicity are important issues in the development of antifungal agents [26]. Clinically available antifungal agents, such as amphotericin B and FLC, are generally suffered from significant side effects [51]. Compounds **A1** and **A2** displayed relatively low toxicity on human normal cell line HUVEC (IC_50_ > 29 μg/mL), which had high selective indexes. Also, no obvious toxicity was observed during the *in vivo* treatments. Thus, the safety profile of the benzoanilide derivatives deserved to be further evaluated.

Virulence factors are produced by fungal pathogens which promote the infectious process and the development drug resistance [52]. The discovery of anti-virulence agents is emerging as a promising strategy to combat antifungal drug resistance [53]. Although the roles of *C. auris* biofilms are less understood than those of biofilms formed by other *Candida* species, *C. auris* biofilms certainly contributed to the severe virulence and resistance [31]. In contrast to weak biofilm inhibition of FLC, compounds **A1** and **A2** could significantly suppress the biofilm formation of *C. auris* and had moderate inhibitory effect for the mature biofilm. *C. auris* was unable to form true hyphae and the underlying reasons are currently unknown [54]. In contrast, compounds **A1** and **A2** completely inhibited the hypha formation of *C. albicans* at the concentration of 0.25 μg/mL. The anti-virulence activity of compounds **A1** and **A2** highlighted the potential of to treat resistant *Candida* infections.

Fungal cell walls are primarily composed of chitin, glucans, mannans and glycoproteins. We found that compound **A1** destroyed the mannan coats and unmasked β-glucan of *C. auris* 0029 cell walls using a microscopic observation assay. The outer layer of cell wall mostly consists of dense mannan coats that are decorated with branched *N*-linked mannan, linear *O*-linked mannan and GPI anchor [55,56]. Recent studies revealed that *Candida* species tended to induce innate immune responses when the outer mannan layer was destroyed [57–59]. Inspired by these observations, a series of mechanism-of-action assays supported the hypothesis that compound **A1** blocked the biosynthesis of virulence factors and fungal cell walls through the inhibition of GPI and GPI-anchored proteins. First, the fluorescence intensity of mannan layer in *C. auris* 0029 cells decreased significantly after the incubation with compound **A1**, while β-glucan was significantly increased by 11.6-fold, which was well consistent with the results from the microscopic observation. Second, TNF-α of mouse macrophages was observed at a significantly higher level in **A1**-treated *C. auris* 0029 cells versus the control group. Third, the FLAER fluorescence intensity of fungal cells was significantly decreased after the treatment of compound **A1**. In addition, compound **A1** significantly down-regulated the expression of *GWT1*, *HWP1*, and *ALS3* genes, which were related to GPI, adhesion, and biofilm, respectively. The Gwt1 inhibitory potency of compound **A1** was further verified by GWT1 knockdown strains. These results suggested that compound **A1** could effectively inhibit the biosynthesis of mannan in drug-resistant *C. auris* cell walls. It also should be noted that target identification of active compounds from phenotypic screening is highly challenging. Further exploration of the mechanism-of-action of the benzoanilide derivatives should be prioritized.

Taken together, novel benzoanilide antifungal lead compounds were identified to treat multi-resistant *C. auris* infections. Phenotypic screening and hit optimization identified compound **A1** with excellent antifungal activity and low cytotoxicity. Compound **A1** exhibited anti-virulence properties to hyphae and biofilm. In particular, it was orally active and could significantly reduce kidney fungal burden in murine models of drug-resistant *C. auris* and *C. albicans*. Interestingly, compound **A1** could inhibit the biosynthesis of GPIs and GPI-anchored proteins by downregulating *GWT1*, *HWP1* and *ALS3* gene, and then blocked the synthesis of mannan in fungal cell walls, resulting in the exposure of β-glucan, damage of fungal cells and the host immune response. Taken together, compound **A1** represents a promising lead compound for treatment of drug resistant candidiasis. Further mechanistic validation, antifungal evaluations, and structural optimizations are currently in progress.

## Supplementary Material

Supplemental Material

Supplemental Material

## Data Availability

All the data including experimental procedures, synthetic methods, structural characterization and spectra data are available in the supplementary information files.
