# Supplementary material for: Discovery of a new chemical scaffold for the treatment of superbug Candida auris infections
Source: Emerg Microbes Infect. 2023 May 11;12(1):2208687. doi: 10.1080/22221751.2023.2208687 (PMC10177697; doi:10.1080/22221751.2023.2208687)
Supplement: Supplemental Material [file TEMI_A_2208687_SM3478.docx]

**Graphical abstract**

**Discovery of a new chemical scaffold for the treatment of superbug *Candida auris* infections**

Jie Tu**^#^**, Tianbao Zhu**^#^**, Qingwen Wang**^#^**, Wanzhen Yang, Yahui Huang, Defeng Xu*, Na Liu*, Chunquan Sheng*


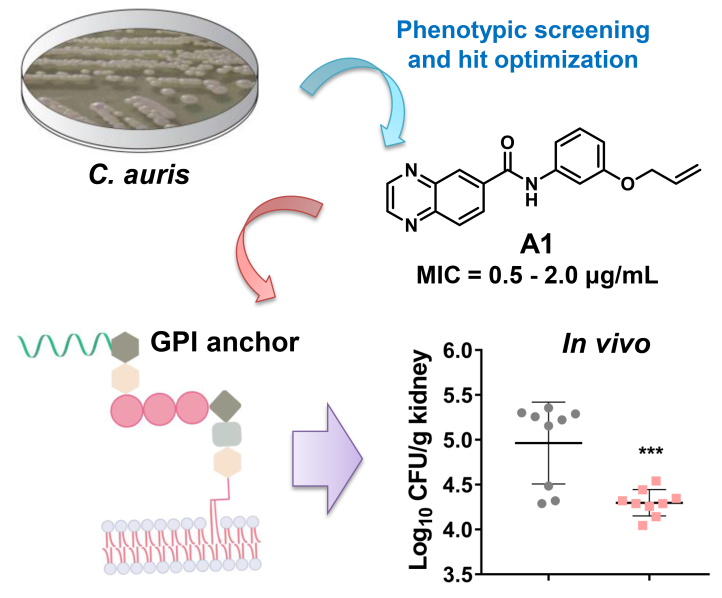


Novel benzoanilide antifungal agents were discovered to treat multi-resistant *C. auris* infections by an integrated strategy of phenotypic screen, hit optimization, antifungal assays and mechanism exploration. The most promising compound **A1** inhibited the biosynthesis of GPIs and GPI-anchored proteins, which showed potent *in vitro* and *in vivo* efficacy against *C. auris* infection.
