# Supplementary material for: Discovery of a new chemical scaffold for the treatment of superbug Candida auris infections
Source: Emerg Microbes Infect. 2023 May 11;12(1):2208687. doi: 10.1080/22221751.2023.2208687 (PMC10177697; doi:10.1080/22221751.2023.2208687)

**Supplementary Material**

**Discovery of a new chemical scaffold for the treatment of superbug *Candida auris* infections**

Jie Tu^1^**^#^**, Tianbao Zhu^1,2^**^#^**, Qingwen Wang^1^**^#^**, Wanzhen Yang^1^, Yahui Huang^1^, Defeng Xu^2^*, Na Liu^1^*, Chunquan Sheng^1^*

*^1^ School of Pharmacy, Second Military Medical University (Naval Medical University), Shanghai 200433, China*;

*^2^ School of Pharmacy, Changzhou University, Changzhou 213164, China*

* Correspondence:

Chunquan Sheng, shengcq@smmu.edu.cn; Na Liu, liuna@smmu.edu.cn; Defeng Xu. markxu@cczu.edu.cn.

**^#^** These authors contributed equally to this work.

**Table of Contents**

[**Table S1** *In vitro* antifungal activities of compounds **A1**-**A15**, **B**1-**B10** and **C1**-**C4** against *Candida auris* (MIC, μg/mL) S3](#_Toc130567343)

[**Table S2** *In vitro* antifungal spectrum of compounds **A1-A15**, **B1-B10** and **C1-C4** (MIC, μg/mL) S4](#_Toc130567344)

[**Table S3** *In vitro* cytotoxicity and anti-resistance fungi activity of compounds **A1**-**A4** and **A14**-**A15** S5](#_Toc130567345)

[**Figure S1**. Mechanism-of-action of compound **A1** on *C. albicans* 103. S6](#_Toc130567346)

[**Table S4** Gene primer sequences of real time RT-PCR assay S6](#_Toc130567347)

[General Synthetic Route S6](#_Toc130567348)

[Chemical Synthesis and Structural Characterization of Compounds S9](#_Toc130567349)

[*In Vitro* Cell Cytotoxicity Evaluation S15](#_Toc130567350)

[Spectral Data S17](#_Toc130567351)

# Table S1 *In vitro* antifungal activities of compounds A1-A15, B1-B10 and C1-C4 against *Candida auris* (MIC, μg/mL)

| **Compds.** | ***C. aur.****^a^* **0029** | ***C. aur.****^a^* **0030** | ***C. aur.****^a^* **15448** |
| --- | --- | --- | --- |
| **A1** | 2.0 | 1.0 | 0.50 |
| **A2** | 0.25 | 0.13 | 0.063 |
| **A3** | 0.13 | 0.13 | 0.031 |
| **A4** | 0.5 | 0.25 | 0.063 |
| **A5** | >64 | >64 | >64 |
| **A6** | >64 | >64 | >64 |
| **A7** | >64 | >64 | >64 |
| **A8** | >64 | >64 | 64 |
| **A9** | >64 | >64 | 8 |
| **A10** | >64 | >64 | >64 |
| **A11** | >64 | >64 | >64 |
| **A12** | >64 | >64 | >64 |
| **A13** | >64 | >64 | >64 |
| **A14** | 0.25 | 0.13 | 0.063 |
| **A15** | 0.50 | 0.25 | 0.50 |
| **B1** | >64 | >64 | >64 |
| **B2** | >64 | >64 | >64 |
| **B3** | >64 | >64 | >64 |
| **B4** | >64 | >64 | >64 |
| **B5** | >64 | >64 | >64 |
| **B6** | >64 | >64 | >64 |
| **B7** | >64 | >64 | >64 |
| **B8** | >64 | >64 | >64 |
| **B9** | >64 | >64 | >64 |
| **B10** | >64 | >64 | >64 |
| **C1** | >64 | >64 | >64 |
| **C2** | >64 | >64 | >64 |
| **C3** | >64 | >64 | >64 |
| **C4** | 8.0 | 8.0 | 2.0 |
| **NMU-6** | 8.0 | 4.0 | 1.0 |
| **FLC***^a^* | >64 | >64 | 4.0 |
| **Caspofungin** | >64 | 64 | 64 |

*^a^Abbreviations*: *C. aur.*, *Candida auris*; FLC, fluconazole*.*

# Table S2 *In vitro* antifungal spectrum of compounds A1-A15, B1-B10 and C1-C4 (MIC, μg/mL)

| **Compds.** | ***C. alb.****^a^*  **SC5314** | ***C. alb.****^a^*  **103** | ***C. tro.****^a^*  **10086** | ***C. neo.****^a^*  **H99** | ***C. gla.****^a^*  **7669** | ***C. lus.****^a^*  **9150** | ***C. par.****^a^*  **20090** |
| --- | --- | --- | --- | --- | --- | --- | --- |
| **A1** | 0.25 | 0.13 | 0.50 | 4.0 | 2.0 | 4.0 | 0.50 |
| **A2** | 0.031 | 0.13 | 0.031 | 1.0 | 4.0 | 4.0 | 0.50 |
| **A3** | 0.031 | 0.063 | 0.031 | 4.0 | 0.50 | 1.0 | 0.031 |
| **A4** | 0.063 | 0.13 | 0.031 | 4.0 | 1.0 | 4.0 | 0.031 |
| **A5** | >64 | >64 | >64 | >64 | >64 | >64 | >64 |
| **A6** | >64 | >64 | >64 | 64 | >64 | >64 | >64 |
| **A7** | >64 | >64 | >64 | 64 | >64 | >64 | >64 |
| **A8** | >64 | >64 | >64 | 8.0 | >64 | >64 | >64 |
| **A9** | 2.0 | 0.13 | >64 | >64 | >64 | >64 | 2.0 |
| **A10** | >64 | 8.0 | >64 | >64 | >64 | >64 | >64 |
| **A11** | >64 | >64 | >64 | 64 | >64 | >64 | >64 |
| **A12** | >64 | >64 | >64 | 32 | >64 | >64 | >64 |
| **A13** | 64 | >64 | 32 | 64 | >64 | >64 | >64 |
| **A14** | 0.031 | 0.031 | 0.031 | 0.063 | 8.0 | 16 | 1.0 |
| **A15** | 0.13 | 0.13 | 0.13 | 0.50 | 2.0 | 4.0 | 1.0 |
| **B1** | >64 | >64 | >64 | >64 | >64 | >64 | >64 |
| **B2** | >64 | >64 | >64 | >64 | >64 | >64 | >64 |
| **B3** | >64 | >64 | >64 | >64 | >64 | >64 | >64 |
| **B4** | >64 | >64 | >64 | >64 | >64 | >64 | >64 |
| **B5** | >64 | >64 | >64 | >64 | >64 | >64 | >64 |
| **B6** | >64 | >64 | >64 | >64 | >64 | >64 | >64 |
| **B7** | 32 | 32 | 64 | 4.0 | >64 | >64 | 32 |
| **B8** | >64 | >64 | >64 | >64 | >64 | >64 | >64 |
| **B9** | >64 | >64 | >64 | >64 | >64 | >64 | >64 |
| **B10** | >64 | >64 | >64 | >64 | >64 | >64 | >64 |
| **C1** | >64 | >64 | >64 | >64 | >64 | >64 | >64 |
| **C2** | >64 | >64 | >64 | 32 | >64 | >64 | >64 |
| **C3** | >64 | >64 | >64 | 64 | >64 | >64 | >64 |
| **C4** | 8.0 | 0.25 | 8.0 | 16 | 16 | 32 | 16 |
| **NMU-6** | 8.0 | 4.0 | 4.0 | 8.0 | 8.0 | 8.0 | 4.0 |
| **FLC** | 0.25 | >64 | >64 | 2.0 | 0.25 | 1.0 | 2.0 |

*^a^Abbreviations*: *C. alb.*, *Candida albicans*; *C. tro.*, *Candida tropicalis*; *C. neo.*, *Cryptococcus neoformans*; *C. gla.*, *Candida glabrata*; *C. lus.*, *Candida lusitaniae*; *C. par.*, *Candida parapsilosis*.

# Table S3 *In vitro* cytotoxicity and anti-resistance fungi activity of compounds A1-A4 and A14-A15

| **Compds**. | Cytotoxicity to HUVEC  (IC_50_, μg/mL) | Anti-resistant *C. alb*. 103  (MIC, μg/mL) |
| --- | --- | --- |
| **A1** | 61 | 0.13 |
| **A2** | 29 | 0.13 |
| **A3** | 18 | 0.063 |
| **A4** | 25 | 0.13 |
| **A14** | 16 | 0.031 |
| **A15** | 14 | 0.13 |


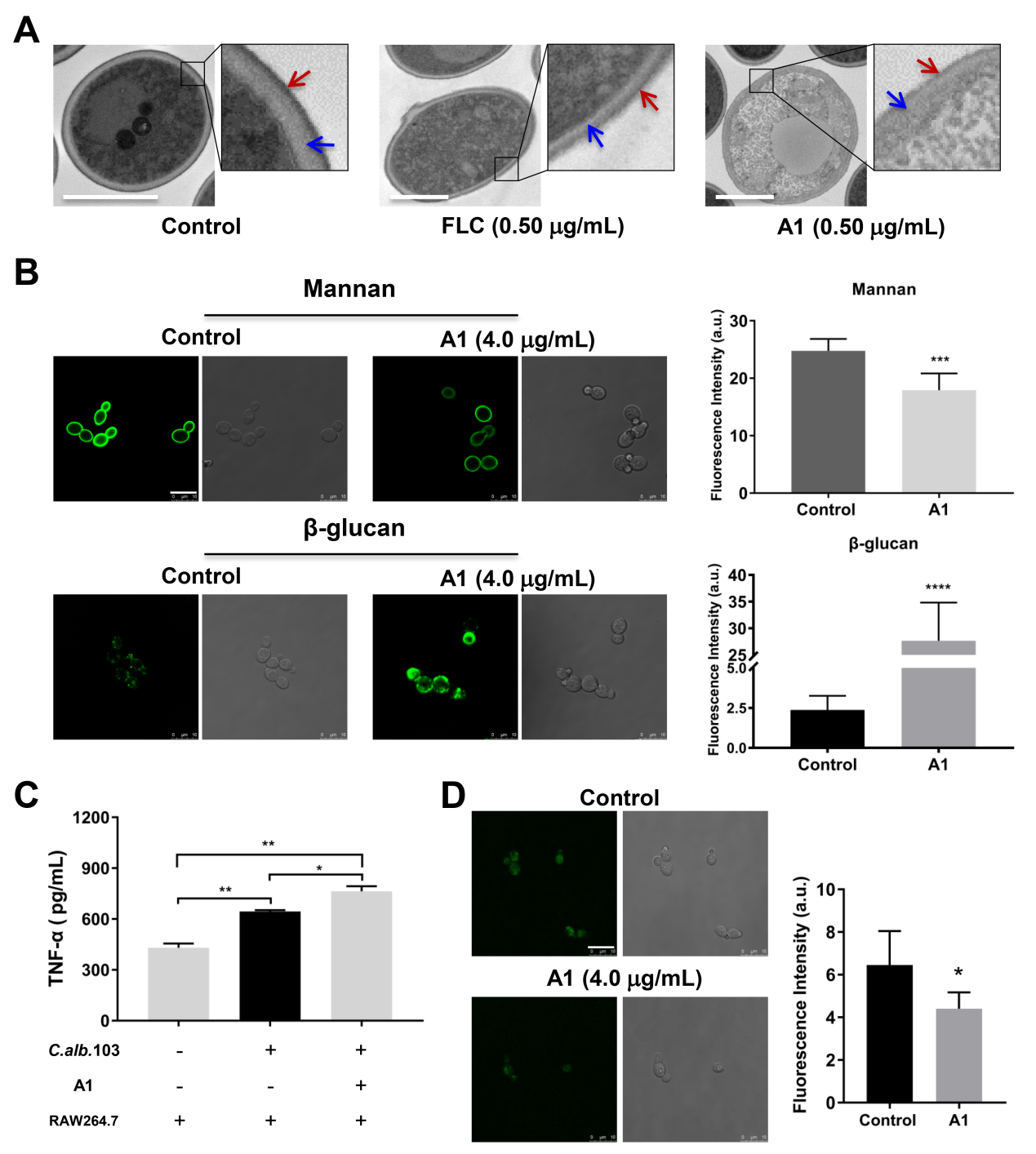


# Figure S1. Mechanism-of-action of compound A1 on *C. albicans* 103.

(A) Transmission electron images of drug-resistant *C. albicans* 103 after incubating with FLC and compound **A1**. The red and blue arrows indicate the mannan coats and β-glucan layer, respectively. Scale bar represents 1 µm. (B) Fluorescence intensity assay of mannan and β-glucan in *C. albicans* 103 cell walls after treatment with compound **A1**. Scale bar represents 10 µm. ****P* < 0.001, and *****P* < 0.0001, vs. the control group, determined by two-tailed unpaired Student’s *t*-test. (C) The TNF-α concentration analysis by co-culture of macrophages RAW264.7 and *C. albicans* 103 treated by compound **A1** (4.0 μg/mL). **P* < 0.05, and ***P* < 0.01, determined by one-way ANOVA. (D) FLAER staining of GPI-anchored proteins in fungal cells. Scale bar represents 10 µm. **P* < 0.05, vs. the control group, determined by two-tailed unpaired Student’s *t*-test.

# Table S4 Gene primer sequences of real time RT-PCR assay

| **Name** | **Sequence** |
| --- | --- |
| *GWT1F* | TCTCAGTCGCCACTACTCAG |
| *GWT1R* | GCATTGTACGAAACGACCCA |
| *HWP1F* | TCCGGAATCTAGTGCTGTCGT |
| *HWP1R* | GCGACACTTGAGTAATTGGCAG |
| *ALS3F* | ACTTTGTGGTCTACAACTTGGG |
| *ALS3R* | CCAGATGGGGATTGTAAAGTGG |
| *ACT1F* | TGACAAATGGGTAGGGTGGGA |
| *ACT1*R | GAAACCACTGCCGACAGATCA |

General Synthetic Route

The chemical synthesis of target compounds is depicted in **Schemes S1-S4**. Intermediates **3a-3h** were synthesized from commercially available nitrophenols (**1a-1c**) and various substituted bromines (**2a-2f**), and then reduced by Fe/NH_4_Cl to obtain intermediates **4a-4h**. Acids **5-6** reacted with intermediates **4a-4h** by the condensation substitution in the presence of 2-(1*H*-benzotriazole-1-yl)-1,1,3,3-tetramethyluronium hexafluorophosphate (HBTU) to form intermediates **7-9** and target compounds **A1-A9**. Compound **A10** was obtained by azide reaction of compound **A4** with chloromethyl benzene. Hydrolysis of intermediate **7** with hydroxylamine hydrochloride solution gave compound **A11**. In the presence of K_2_CO_3_ and *N*,*N*-dimethylformamide (DMF), intermediate **8** was substituted with commercially available (*R*)-pyrrolidin-3-ylmethanol to obtain target compound **A12**. Intermediate **9** was deprotected by pyridine *p*-toluene sulfonates (PPTs) and methanol to afford compound **A13** (**Scheme S1**). By similar synthetic protocols described in **Scheme S1**, target compounds **A14**-**A15** were obtained from 3-nitrophenol and trimethylchlorosilane via reduction and substitution reactions (**Scheme S2**).

**Scheme S1** Reagents and condition: (**a**) K_2_CO_3_, CH_3_COCH_3_, r.t, yield 89%; (**b**) Fe, NH_4_Cl, EtOH, H_2_O, reflux, yield 93%; (**c**) HBTU, Et_3_N, DMF, r.t, yields 39~82%; (**d**) Chloromethyl benzene, Cu(OAc)_2_, NaN_3_, EtOH, H_2_O, *L*-sodium ascorbate, 1,10-phenanthroline, r.t, yield 46%; (**e**) NH_4_Cl, KOH, MeOH, r.t; yield 10%; (**f**) (*R*)-Pyrrolidin-3-ylmethanol, K_2_CO_3_, DMF, 60 °C, yield 47%; (**g**) PPTs, CH_3_OH, r.t, yield 40%.

**Scheme S2** Reagents and condition: (**a**) Imidazole, trimethylchlorosilane, DMF, r.t, yield 93%; (**b**) H_2_, Pd/C, EtOH, r.t, yield 83%; (**c**) HBTU, Et_3_N, DMF, r.t, yields 56-73%; (**d**) LiOH, DMF, r.t, yields 82-84%; (**e**) Bromomethyl cyclopropane, K_2_CO_3_, EtOH, reflux, yields 45-62%.

Intermediate **4a** was synthesized according to the method depicted in **Scheme S1**. Then various substituted carboxylic acids reacted with intermediate **4a** in the presence of HBTU to give target compounds **B1-B7**. Compounds **B8**-**B10** were synthesized by the condense reaction between intermediate **4a** and substituted sulfonyl chlorides or peroxyacyl chloride (**Scheme S3**).

**Scheme S3** Reagents and condition: (**a**) HBTU, Et_3_N, DMF, r.t, yields 35~62%; (**b**) Pyridine, N_2_, DCM, reflux, yields 39~41%; (**c**) EA, THF, H_2_O, Na_2_CO_3_, 0 °C, yield 27%.

Intermediate **18** was synthesized by the demethylation of intermediate **17**, which was obtained by condensation of 6-aminoquinoline with 3-methoxyphenyl isocyanate, and then substituted by allyl bromine to give target compound **C1**. Intermediate **21** was obtained from quinoxaline benzoate via esterification and ammonolysis, and reacted by nucleophilic addition reaction with intermediates **22-23** to form compounds **C2-C3**. The reaction of m-diphenol with bromomethyl cyclopropane gave intermediate **25**. Intermediate **27** was obtained by the reaction of compounds **26** with chloroacetyl chloride, which was finally reacted with intermediate **25** to afford target compounds **C4** (**Scheme S4**).

**Scheme S4** Reagents and condition: (**a**) 3-Methoxyphenyl isocyanate, DCM, r.t, yield 98%; (**b**) HBr/CH_3_COOH, 70 °C, yield 74%; (**c**) Allyl bromide, K_2_CO_3_, EtOH, reflux, yield 37%; (**d**) SOCl_2_, MeOH, r.t, yield 95%; (**e**) NH_2_-NH_2_**·**H_2_O, MeOH, reflux, yield 63%; (**f**) CH_3_COOH, MeOH, r.t, yield 72-75%; (**g**) Bromomethyl cyclopropane, K_2_CO_3_, EtOH, reflux, yield 38%; (**h**) Chloroacetyl chloride, DIPEA, THF, 0 °C, yields 65-78%; (**i**) K_2_CO_3_, EtOH, 85 °C, yields 77-86%.

Chemical Synthesis and Structural Characterization of Compounds

***General methods****.* The ^1^H NMR and ^13^C NMR spectra of the target compounds were tested on Bruker AVANCE 300 or AVANCE 600 spectrometer (Bruker Company, Germany), using TMS as internal standard and CDCl_3_ or DMSO-*d_6_* as the solvent. Chemical reactions were monitored by thin-layer chromatography (TLC), with silica gel GF254 as the stationary phase. High-resolution mass spectrometry (HRMS) tests were performed on an Agilent UPLC-QTOF/MS mass spectrometer. The purity analysis of the target product was completed on Agilent C18 with CH_3_OH/H_2_O (V/V, 8/2) as mobile phase (0.5 mL/min flow rate). Purities of all final compounds show greater than 95%.

***N*-(3-(Allyloxy)phenyl)quinoxaline-6-carboxamide (A1)**. Intermediate **4a** (0.20 g, 1.3 mmol) and quinoxaline-6-carboxylic acid (0.22 g, 1.2 mmol) were dissolved in DMF and stirred at room temperature. The condensation agent HBTU (0.56 g, 1.5 mmol) and triethylamine (0.27 g, 2.7 mmol) were added to the mixture and stirred overnight. After the reaction, water (30 mL) was added for dilution and EtOAc (50 mL) extraction, and then the organic phase was washed with saturated NaCl solution (4 × 30 mL), dried with anhydrous Na_2_SO_4_, concentrated and column chromatography to obtain target compound **A1** (0.16 g, yield 57.4%) as yellow solid. ^1^H NMR (600 MHz, DMSO) δ: 10.58 (s, 1H), 9.06 (dd, *J* = 9.7, 1.8 Hz, 2H), 8.75 (d, *J* = 1.9 Hz, 1H), 8.35 (dd, *J* = 8.7, 2.0 Hz, 1H), 8.23 (d, *J* = 8.7 Hz, 1H), 7.56 (t, *J* = 2.2 Hz, 1H), 7.45*-*7.41 (m, 1H), 7.28 (t, *J* = 8.1 Hz, 1H), 6.74 (ddd, *J* = 8.2, 2.5, 0.7 Hz, 1H), 6.12*-*6.02 (m, 1H), 5.45*-*5.39 (m, 1H), 5.31*-*5.25 (m, 1H), 4.59*-*4.56 (m, 2H). ^13^C NMR (151 MHz, DMSO) δ: 164.9, 158.8, 147.5, 147.2, 143.9, 141.9, 140.5, 136.4, 134.1, 129.9, 129.3, 129.1, 117.8, 113.2, 110.6, 107.4, 68.6. HRMS: calculated for C_18_H_15_N_3_O_2_ ([M+H]^+^) 306.1237, found 306.1240.

The synthetic methods of target compounds **A1-A9** refer to compound **A1**.

***N*-(3-(Allyloxy)phenyl)quinoline-6-carboxamide (A2)**. Light yellow solid, 0.089 g, yield 45.8%. ^1^H NMR (600 MHz, DMSO) δ: 10.44 (s, 1H), 8.99 (dd, *J* = 4.2, 1.7 Hz, 1H), 8.60 (d, *J* = 2.0 Hz, 1H), 8.52-8.50 (m, 1H), 8.22 (dd, *J* = 8.8, 2.0 Hz, 1H), 8.11 (d, *J* = 8.8 Hz, 1H), 7.62 (dd, *J* = 8.3, 4.2 Hz, 1H), 7.51 (t, *J* = 2.2 Hz, 1H), 7.39-7.37 (m, 1H), 7.25 (t, *J* = 8.1 Hz, 1H), 6.72-6.69 (m, 1H), 6.07-6.01 (m, 1H), 5.41-5.37 (m, 1H), 5.26-5.24 (m, 1H), 4.56-4.54 (m, 2H). ^13^C NMR (151 MHz, DMSO) δ: 164.9, 158.8, 147.5, 147.2, 143.9, 141.9, 140.5, 136.4, 134.1, 129.9, 129.3, 129.1, 117.8, 113.2, 110.6, 107.4, 68.6. HRMS: calculated for C_19_H_16_N_2_O_2_ ([M+H]^+^) 305.1285, found 305.1281.

***N*-(4-(Allyloxy)phenyl)quinoline-6-carboxamide (A3)**. White solid, 0.12 g, yield 62.1%. ^1^H NMR (600 MHz, DMSO) δ: 10.48 (s, 1H), 9.02 (dd, *J* = 4.2, 1.7 Hz, 1H), 8.62 (d, *J* = 2.0 Hz, 1H), 8.57-8.50 (m, 1H), 8.25 (dd, *J* = 8.8, 2.0 Hz, 1H), 8.14 (d, *J* = 8.7 Hz, 1H), 7.65 (q, *J* = 12 Hz, 1H), 7.54 (t, *J* = 2.2 Hz, 1H), 7.41-7.40 (m, 1H), 7.28 (t, *J* = 8.1 Hz, 1H), 6.79-6.67 (m, 1H), 6.10-6.04 (m, 1H), 5.44-5.40 (m, 1H), 5.29-5.26 (m, 1H), 4.58 (m, 2H). ^13^C NMR (151 MHz, DMSO) δ: 164.9, 158.8, 147.5, 147.2, 143.9, 141.9, 140.5, 136.4, 134.1, 129.9, 129.3, 129.1, 117.8, 113.2, 110.6, 107.4, 68.6. HRMS: calculated for C_19_H_16_N_2_O_2_ ([M+H]^+^) 305.1285, found 305.1291.

***N*-(3-(Prop-2-yn-1-yloxy)phenyl)quinoline-6-carboxamide (A4)**. White solid, 0.21 g, yield 72%. ^1^H NMR (600 MHz, DMSO) δ: 10.53 (s, 1H), 9.03 (dd, *J* = 4.2, 1.7 Hz, 1H), 8.64 (d, *J* = 2.0 Hz, 1H), 8.59-8.52 (m, 1H), 8.27 (dd, *J* = 8.8, 2.1 Hz, 1H), 8.16 (d, *J* = 8.8 Hz, 1H), 7.65 (dd, *J* = 8.3, 4.2 Hz, 1H), 7.58 (t, *J* = 2.2 Hz, 1H), 7.50-7.45 (m, 1H), 7.32 (t, *J* = 8.2 Hz, 1H), 6.81-6.78 (m, 1H), 4.82 (d, *J* = 2.4 Hz, 2H), 3.59 (t, *J* = 2.4 Hz, 1H). ^13^C NMR (151 MHz, DMSO) δ: 165.6, 157.9, 152.7, 149.2, 140.7, 137.6, 133.2, 129.9, 129.5, 128.9, 128.5, 127.5, 122.7, 113.8, 110.4, 107.6, 79.7, 78.6, 55.9. HRMS: calculated for C_19_H_14_N_2_O_2_ ([M+H]^+^) 303.1128, found 303.1122.

***N*-(3-((2-(Methylcarbamoyl)pyridin-4-yl)oxy)phenyl)quinoline-6-carboxamide (A5)**. White solid, 0.29 g, yield 81.2%. ^1^H NMR (600 MHz, DMSO) δ: 10.71 (s, 1H), 9.03 (dd, *J* = 4.2, 1.7 Hz, 1H), 8.82-8.77 (m, 1H), 8.65 (d, *J* = 2.0 Hz, 1H), 8.57 (d, *J* = 5.6 Hz, 1H), 8.56-8.54 (m, 1H), 8.26 (dd, *J* = 8.8, 2.0 Hz, 1H), 8.15 (d, *J* = 8.8 Hz, 1H), 7.80-7.78 (m, 2H), 7.66 (dd, *J* = 8.3, 4.2 Hz, 1H), 7.55 (t, *J* = 8.4 Hz, 1H), 7.48 (d, *J* = 2.6 Hz, 1H), 7.25 (dd, *J* = 5.6, 2.6 Hz, 1H), 7.05-6.98 (m, 1H), 2.81 (d, *J* = 4.9 Hz, 3H). ^13^C NMR (151 MHz, DMSO) δ: 165.8, 164.2, 154.0, 153.0, 152.8, 151.0, 149.2, 141.6, 137.6, 132.9, 131.1, 129.6, 129.0, 128.4, 127.5, 122.7, 117.8, 116.3, 114.9, 112.7, 109.7, 26.4. HRMS: calculated for C_23_H_18_N_4_O_3_ ([M+H]^+^) 399.1457, found 399.1438.

***N*-(2-(Allyloxy)phenyl)quinoxaline-6-carboxamide (A6)**. Light yellow solid, 0.078 g, yield 39.6%. ^1^H NMR (600 MHz, DMSO) δ: 9.95 (s, 1H), 9.06 (dd, *J* = 8.1, 1.8 Hz, 2H), 8.72 (s, 1H), 8.35 (dd, *J* = 8.7, 1.9 Hz, 1H), 8.23 (d, *J* = 8.7 Hz, 1H), 7.74 (dd, *J* = 7.8, 1.3 Hz, 1H), 7.38-6.89 (m, 3H), 6.10-6.03 (m, 1H), 5.46-5.41 (m, 1H), 5.25-5.21 (m, 1H), 4.68-4.64(m, 2H). ^13^C NMR (151 MHz, DMSO) δ: 164.6, 151.6, 147.5, 147.2, 143.9, 142.0, 136.2, 134.1, 130.0, 129.1, 129.1, 127.3, 126.7, 125.8, 120.9, 117.5, 113.5, 69.2. HRMS: calculated for C_18_H_15_N_3_O_2_ ([M+H]^+^) 306.1243, found 306.1246.

***N*-(2-(Allyloxy)phenyl)quinoline-6-carboxamide (A7)**. Yellow solid, 0.17 g, yield 45.7%. ^1^H NMR (600 MHz, DMSO) δ: 9.74 (s, 1H), 9.02 (dd, *J* = 4.2, 1.7 Hz, 1H), 8.65 (d, *J* = 1.7 Hz, 1H), 8.53 (d, *J* = 7.6 Hz, 1H), 8.26 (dd, *J* = 8.8, 1.9 Hz, 1H), 8.14 (d, *J* = 8.8 Hz, 1H), 7.77 (dd, *J* = 7.8, 1.3 Hz, 1H), 7.64 (dd, *J* = 8.3, 4.2 Hz, 1H), 7.22*-*7.17 (m, 1H), 7.12 (d, *J* = 7.3 Hz, 1H), 7.00 (td, *J* = 7.7, 1.1 Hz, 1H), 6.12*-*6.01 (m, 1H), 5.48*-*5.40 (m, 1H), 5.28*-*5.20 (m, 1H), 4.69*-*4.64 (m, 2H). ^13^C NMR (151 MHz, DMSO) δ: 165.1, 152.7, 151.2, 149.2, 137.6, 134.1, 132.8, 129.6, 128.8, 128.2, 127.6, 127.5, 126.4, 125.3, 122.7, 120.9, 117.5, 113.4, 69.2. HRMS: calculated for C_19_H_16_N_2_O_2_ ([M+H]^+^) 305.1290, found 305.1291.

***N*-(4-(Allyloxy)phenyl)quinoxaline-6-carboxamide (A8)**. White solid, 0.17 g, yield 69.7%. ^1^H NMR (600 MHz, DMSO) δ: 10.62 (s, 1H), 9.08 (dd, *J* = 9.7, 1.7 Hz, 2H), 8.76 (d, *J* = 1.9 Hz, 1H), 8.36 (dd, *J* = 8.7, 2.0 Hz, 1H), 8.24 (d, *J* = 8.7 Hz, 1H), 7.57 (t, *J* = 2.1 Hz, 1H), 7.47-7.42 (m, 1H), 7.29 (t, *J* = 8.1 Hz, 1H), 6.77-6.73 (m, 1H), 6.12-6.05 (m, 1H), 5.45-5.41 (m, 1H), 5.30-5.37 (m, 1H), 4.59 (d, *J* = 5.2 Hz, 2H). ^13^C NMR (151 MHz, DMSO) δ: 164.4, 155.1, 147.5, 147.2, 143.8, 142.0, 136.5, 134.3, 132.6, 129.8, 129.3, 129.0, 122.5, 117.8, 115.1, 68.8. HRMS: calculated for C_18_H_15_N_3_O_2_ ([M+H]^+^) 306.1243, found 306.1229.

***N*-(3-(Prop-2-yn-1-yloxy)phenyl)quinoxaline-6-carboxamide (A9)**. White solid, 0.16 g, yield 58.8%. ^1^H NMR (600 MHz, DMSO) δ: 10.64 (s, 1H), 9.08 (d, *J* = 1.8 Hz, 1H), 9.06 (d, *J* = 1.8 Hz, 1H), 8.75 (d, *J* = 2.0 Hz, 1H), 8.35 (dd, *J* = 8.7, 2.0 Hz, 1H), 8.24 (d, *J* = 8.7 Hz, 1H), 7.58 (d, *J* = 2.2 Hz, 1H), 7.50-7.46 (m, 1H), 7.32 (t, *J* = 8.2 Hz, 1H), 6.80-6.78 (m, 1H), 4.82 (d, *J* = 2.4 Hz, 2H), 3.59 (t, *J* = 2.4 Hz, 1H). ^13^C NMR (151 MHz, DMSO) δ: 164.5, 157.5, 147.1, 146.8, 143.5, 141.5, 140.1, 135.9, 129.5, 129.5, 128.9, 128.7, 113.5, 110.2, 107.3, 79.3, 78.2, 55.5. HRMS: calculated for C_18_H_13_N_3_O_2_ ([M+H]^+^) 304.1086, found 304.1093.

***N*-(3-((1-Benzyl-1*H*-1,2,3-triazol-4-yl)methoxy)phenyl)quinoline-6-carboxamide (A10)**. Copper acetate (0.0031 g, 0.017 mmol), 1,10-phenoline (0.0031 g, 0.017 mmol) and sodium l-asapoate (0.065 g, 0.33 mmol) in a mixed solvent CH_3_OH/H_2_O (v/v, 4/1) were stirred for 5 min, then compound **A4** (0.10 g, 0.33 mmol), benzoic chloride (0.046 g, 0.36 mmol) and sodium azide (0.024 g, 0.36 mmol) were added to stir the reaction at room temperature. At the end of the reaction, EtOAc (3 × 20 mL) was added for extraction, saturated NaCl solution (20 mL) was washed, organic phase was collected, anhydrous Na_2_SO_4_ was dried, and the target product **A10** (0.067 g, yield 46.5%) was reduced by column chromatography as white solid. ^1^H NMR (600 MHz, DMSO) δ: 10.51 (s, 1H), 9.04 (dd, *J* = 4.2, 1.7 Hz, 1H), 8.64 (d, *J* = 2.0 Hz, 1H), 8.56 (dd, *J* = 8.3, 1.2 Hz, 1H), 8.33 (s, 1H), 8.27 (dd, *J* = 8.8, 2.0 Hz, 1H), 8.16 (d, *J* = 8.8 Hz, 1H), 7.67 (dd, *J* = 8.3, 4.2 Hz, 1H), 7.60 (t, *J* = 2.2 Hz, 1H), 7.45 (dd, *J* = 8.1, 1.1 Hz, 1H), 7.42*-*7.38 (m, 2H), 7.37*-*7.33 (m, 3H), 7.32 (t, *J* = 8.2 Hz, 1H), 6.87*-*6.83 (m, 1H), 5.65 (s, 2H), 5.17 (s, 2H).^13^C NMR (151 MHz, DMSO) δ: 165.6, 158.6, 152.7, 149.2, 143.4, 140.7, 137.6, 136.4, 133.2, 129.9, 129.5, 129.2, 128.8, 128.6, 128.5, 128.4, 125.1, 122.7, 113.4, 110.4, 107.5, 61.6, 53.3. HRMS: calculated for C_26_H_21_N_5_O_2_ ([M+H]^+^) 436.1773, found 436.1796.

The synthetic methods of intermediate **7-9** refer to compound **A1**.

***N*-(3-(3-(Hydroxyamino)-3-oxopropoxy)phenyl)quinoline-6-carboxamide (A11)**. Methyl 3-(3-aminophenoxy) propionate reacted with 6-quinoline formic acid to give an intermediate **7** (0.21 g, yield 48.1%) as white solid. Ammonium chloride (0.079 g, 1.1 mmol) and intermediate **7** (0.20 g, 0.57 mmol) were dissolved in methanol, and then potassium hydroxide (0.064 g, 1.1 mmol) was added slowly and reacted for 1 h at room temperature. At the end of the reaction, the reaction liquid was white suspension, vacuum extraction and filtration, filtrate was collected, pH was changed to acidic with acetic acid, solid precipitation, extraction and filtration and washing with water to get the target compound **A11** (0.021 g, yield 10.5%) as white solid. ^1^H NMR (600 MHz, DMSO) δ: 10.55 (s, 1H), 10.41 (s, 1H), 9.02 (dd, *J* = 4.2, 1.7 Hz, 1H), 8.86 (s, 1H), 8.63 (d, *J* = 1.8 Hz, 1H), 8.57*-*8.52 (m, 1H), 8.27 (dd, *J* = 8.8, 2.0 Hz, 1H), 8.14 (d, *J* = 8.8 Hz, 1H), 7.75*-*7.71 (m, 2H), 7.66 (dd, *J* = 8.3, 4.2 Hz, 1H), 6.98*-*6.93 (m, 2H), 4.19 (t, *J* = 6.0 Hz, 2H), 2.44 (t, *J* = 6.0 Hz, 2H). ^13^C NMR (151 MHz, DMSO) δ: 167.0, 165.0, 155.1, 152.4, 148.7, 138.0, 133.4, 132.7, 129.2, 128.7, 128.7, 127.6, 122.7, 122.4, 114.8, 64.3, 33.0. HRMS: calculated for C_19_H_17_N_3_O_4_ ([M+H]^+^) 352.1297, found 352.1303.

**(*S*)-*N*-(3-(2-(2-(Hydroxymethyl)pyrrolidin-1-yl)ethoxy)phenyl)quinoline-6-carboxamide (A12)**. 3-(2-bromoethoxy) aniline with 6-quinoline formic acid gave intermediate **8** (0.098 g, yield 42.6%) as white solid. The intermediates **8** (0.070 g, 0.19 mmol) and (*S*)-(+)-2-pyrrolidine methanol (0.020 g, 0.19 mmol) and potassium carbonate (0.052 g, 0.38 mmol) were dissolved in DMF and reacted at 56 °C. After the reaction, EtOAc (30 mL) was added for extraction, saturated NaCl solution (3 × 20 mL) was washed, organic phase was collected, anhydrous Na_2_SO_4_ was dried, decompression concentration was performed to obtain target compound **A12** (0.035 g, yield 47.3%) as white solid. ^1^H NMR (300 MHz, DMSO) δ: 10.50 (s, 1H), 9.04 (dd, *J* = 4.2, 1.7 Hz, 1H), 8.65 (d, *J* = 2.0 Hz, 1H), 8.58-8.54 (m, 1H), 8.27 (dd, *J* = 8.8, 2.0 Hz, 1H), 8.16 (d, *J* = 8.8 Hz, 1H), 7.67 (dd, *J* = 8.3, 4.2 Hz, 1H), 7.56 (s, 1H), 7.45-7.40 (m, 1H), 7.30 (t, *J* = 8.1 Hz, 1H), 6.74 (dd, *J* = 8.2, 1.9 Hz, 1H), 4.54 (s, 1H), 4.12 (s, 2H), 3.21 (s, 1H), 2.93-2.58 (m, 2H), 2.52-2.34 (m, 2H), 1.95-1.52 (m, 4H), 1.37-1.21 (m, 2H). HRMS: calculated for C_23_H_25_N_3_O_3_ ([M+H]^+^) 392.1896, found 392.1907.

***N*-(3-(2-Hydroxyethoxy)phenyl)quinoline-6-carboxamide (A13)**. 3-(2-((tetrahydro-2*H*-pyran-2-yl)oxy)ethoxy)aniline reacted with 6-quinoline formic acid to obtain yellow solid intermediate **9** (0.46 g, yield 84.1%). The intermediate **9** (0.20 g, 0.51 mmol) and 4-methylbenzenesulfonic acid (0.030 g, 0.12 mmol) were dissolved in methanol solution and stirred at room temperature. After the reaction, part of methanol solution was concentrated under pressure, then water (30 mL) was added to dilute the concentrated solution, and EtOAc (2 × 25 mL) was used for extraction. Organic phase was collected, anhydrous Na_2_SO_4_ was dried, and concentrated under pressure and analyzed by column chromatography to obtain the target compound **A13** (0.063 g, yield 40.1%) as white solid. ^1^H NMR (600 MHz, DMSO) δ: 10.48 (s, 1H), 9.03 (dd, *J* = 4.2, 1.7 Hz, 1H), 8.64 (d, *J* = 1.9 Hz, 1H), 8.57-8.54 (m, 1H), 8.26 (dd, *J* = 8.8, 2.0 Hz, 1H), 8.15 (d, *J* = 8.8 Hz, 1H), 7.66 (dd, *J* = 8.3, 4.2 Hz, 1H), 7.54 (t, *J* = 2.2 Hz, 1H), 7.43-7.39 (m, 1H), 7.28 (t, *J* = 8.1 Hz, 1H), 6.74-6.71 (m, 1H), 4.91 (s, 1H), 4.00 (t, *J* = 5.1 Hz, 2H), 3.79-3.71 (m, 2H). ^13^C NMR (151 MHz, DMSO) δ: 165.6, 159.3, 152.7, 149.2, 140.7, 137.6, 133.2, 129.9, 129.5, 128.8, 128.5, 127.5, 122.7, 113.0, 110.3, 107.1, 69.9, 60.0. HRMS: calculated for C_18_H_16_N_2_O_3_ ([M+H]^+^) 309.1239, found 309.1244.

***N*-(3-(Cyclopropylmethoxy)phenyl)quinoline-6-carboxamide (A14)**. 3-nitrophenol (1.1 g, 7.1 mmol), imidazole (0.73 g, 11 mmol) and trimethylchlorosilane (1.3 g, 8.5 mmol) were dissolved in DMF and stirred at room temperature. After the reaction, 2 M dilute hydrochloric acid was added to adjust PH to acidity, and the organic phase was extracted with EtOAc (2 × 30 mL). The organic phase was collected, washed with saturated NaCl solution for 3 times, dried with anhydrous Na_2_SO_4_, and condensed under pressure to obtain yellow solid intermediate **10** (1.1 g, yield 55%). The intermediate **10** (1.1 g, 3.9 mmol) and 10% palladium carbon (0.031 g) were dissolved in ethanol solution and reacted in a hydrogen atmosphere at room temperature. After the reaction, the palladium carbon was filtered out, and the filter cake was cleaned with EtOAc (2 × 30 mL) to collect the organic phase, which was reduced to a black oil intermediate **11** (0.84 g, yield 95.5%). Intermediate **11** reacted with 6-quinoline formic acid to obtain yellow solid intermediate **12** (0.21 g, yield 45.8%). The intermediate **12** (0.20 g, 0.53 mmol) and lithium hydroxide (0.068 g, 1.6 mmol) were dissolved in DMF and stirred at room temperature. After the reaction, 1M dilute hydrochloric acid was added to the reaction solution to adjust PH to acidity, and yellow solid intermediate **14** (0.12 g, yield 89.2%) was precipitated out of the reaction solution. Intermediates **14** (0.12 g, 0.47 mmol), bromomethyl cyclopropane (0.31 g, 2.3 mmol) and potassium carbonate (0.39 g, 2.9 mmol) were dissolved in DMF and stirred at room temperature. After the reaction, filtered, extracted with EtOAc (30 mL) and water (20 mL), washed with saturated aqueous NaCl solution (2 × 20 mL), organic phase was collected, anhydrous Na_2_SO_4_ was dried, concentrated under pressure and analyzed by column chromatography to obtain the target compound **A14** (0.021 g, yield 13.4%) as white solid. ^1^H NMR (600 MHz, DMSO) δ: 10.45 (s, 1H), 9.01 (d, *J* = 4.2 Hz, 1H), 8.62 (s, 1H), 8.54 (d, *J* = 8.4 Hz, 1H), 8.25 (dd, *J* = 8.7, 1.7 Hz, 1H), 8.14 (d, *J* = 8.8 Hz, 1H), 7.65 (dd, *J* = 8.3, 4.1 Hz, 1H), 7.50 (s, 1H), 7.39 (d, *J* = 8.2 Hz, 1H), 7.26 (t, *J* = 8.1 Hz, 1H), 6.80-6.66 (m, 1H), 3.82 (d, *J* = 6.9 Hz, 2H), 1.23-1.22 (m, 1H), 0.60-0.56 (m, 2H), 0.36-0.31 (m, 2H). ^13^C NMR (151 MHz, DMSO) δ: 165.7, 159.2, 152.7, 149.2, 140.7, 137.6, 133.2, 129.8, 129.5, 128.8, 128.4, 127.5, 122.7, 112.8, 110.5, 107.0, 72.4, 10.6, 3.5. HRMS: calculated for C_20_H_18_N_2_O_2_ ([M+H]^+^) 319.1441, found 319.1451.

The synthetic methods of target compounds **A15** refer to compound **A14**.

***N*-(3-(Cyclopropylmethoxy)phenyl)quinoxaline-6-carboxamide** **(A15**). White solid, 0.03 g, yield 21.6%. ^1^H NMR (300 MHz, DMSO) δ: 10.59 (s, 1H), 9.07 (d, *J* = 3.2 Hz, 2H), 8.75 (d, *J* = 1.7 Hz, 1H), 8.35 (dd, *J* = 8.8, 1.9 Hz, 1H), 8.24 (d, *J* = 8.7 Hz, 1H), 7.52 (s, 1H), 7.42 (d, *J* = 8.2 Hz, 1H), 7.27 (t, *J* = 8.1 Hz, 1H), 6.71 (dd, *J* = 8.1, 2.0 Hz, 1H), 3.83 (d, *J* = 7.0 Hz, 2H), 1.24-1.21 (m, 1H), 0.62-0.54 (m, 2H), 0.37-0.31 (m, 2H). ^13^C NMR (151 MHz, DMSO) δ: 165.6, 157.9, 152.7, 149.2, 140.7, 137.6, 133.2, 129.9, 129.5, 128.9, 128.5, 127.5, 122.7, 113.8, 110.4, 107.6, 79.7, 78.6, 55.9. HRMS: calculated for C_19_H_17_N_3_O_2_ ([M+H]^+^) 320.1394, found 320.1411.

The synthetic methods of target compounds **B1-B7** refer to compound **A1**.

***N*-(3-Allylphenyl)-4-methyl-3-nitrobenzamide (B1).** White solid, 0.16 g, yield 40.3%. ^1^H NMR (600 MHz, CDCl_3_) δ: 8.41 (s, 1H), 7.74 (d, *J* = 12 Hz, 1H), 7.57 (d, *J* = 1.5 Hz, 1H), 7.40 (s, 1H), 7.38-7.35 (m, 1H), 7.26-7.22 (m, 1H), 7.16-7.13 (m, 1H), 6.76-6.73 (m, 1H), 6.07-6.00 (m, 1H), 5.42-5.38 (m, 1H), 5.31-5.27 (m, 1H), 4.53 (dt, *J* = 5.3, 1.5 Hz, 2H), 3.91 (s, 3H). ^13^C NMR (151 MHz, DMSO) δ: 163.8, 158.3, 151.5, 140.9, 140.0, 139.7, 133.6, 129.4, 124.8, 119.6, 117.3, 113.5, 112.9, 110.3, 107.0, 68.1, 56.9. HRMS: calculated for C_17_H_16_N_2_O_5_ ([M+H]^+^) 329.1137, found 329.1144.

***N*^1^,*N*^3^-Bis(3-(allyloxy)phenyl)isophthalamide** (**B2**). Light yellow solid, 0.11 g, yield 38.9%. ^1^H NMR (600 MHz, DMSO) δ: 10.34 (s, 2H), 8.47 (s, 1H), 8.10 (dd, *J* = 7.7, 1.8 Hz, 2H), 7.67 (t, *J* = 12 Hz, 1H), 7.49 (t, *J* = 2.2 Hz, 2H), 7.37-7.34 (m, 2H), 7.24 (t, *J* = 8.1 Hz, 2H), 6.72-6.67 (m, 2H), 6.08-6.00 (m, 2H), 5.41-5.37 (m, 2H), 5.26-5.23 (m, 2H), 4.54 (dt, *J* = 5.2, 1.5 Hz, 4H). ^13^C NMR (151 MHz, DMSO) δ: 165.5, 158.8, 140.7, 135.6, 134.1, 131.1, 129.8, 129.0, 127.4, 117.8, 113.2, 110.4, 107.3, 68.6. HRMS: calculated for C_26_H_24_N_2_O_4_ ([M+H]^+^) 429.1809, found 429.1830.

**(*E*)-*N*-(3-(Allyloxy)phenyl)-3-(4-methoxyphenyl)acrylamide** (**B3**). White solid, 0.09 g, yield 35.1%. ^1^H NMR (600 MHz, DMSO) δ: 10.14 (s, 1H), 7.53 (d, *J* = 15.7 Hz, 1H), 7.44-7.41 (m, 1H), 7.33 (t, *J* = 7.9 Hz, 1H), 7.22-7.13 (m, 4H), 6.96 (ddd, *J* = 8.2, 2.6, 0.7 Hz, 1H), 6.79 (d, *J* = 15.7 Hz, 1H), 6.66-6.62 (m, 1H), 6.07-5.98 (m, 1H), 5.41-5.35 (m, 1H), 5.27-5.22 (m, 1H), 4.52 (dt, *J* = 5.2, 1.5 Hz, 2H), 3.78 (s, 3H). ^13^C NMR (151 MHz, DMSO) δ: 163.9, 160.1, 158.9, 140.8, 140.6, 136.6, 134.1, 130.5, 130.0, 123.0, 120.5, 117.9, 116.1, 113.1, 112.1, 109.9, 106.3, 68.6, 55.6. HRMS: calculated for C_19_H_19_NO_3_ ([M+H]^+^) 310.1443, found 310.1435.

***N*-(3-(Allyloxy)phenyl)-4-ethynylbenzamide (B4)**. White solid, 0.11 g, yield 53.1%. ^1^H NMR (600 MHz, DMSO) δ: 10.21 (s, 1H), 7.90-7.88 (m, 2H), 7.58-7.56 (d, *J* = 8.4 Hz, 2H), 7.42 (t, *J* = 12 Hz, 1H), 7.30-7.28 (m, 1H), 7.18 (t, *J* = 18 Hz 1H), 6.65 (ddd, *J* = 8.2, 2.5, 0.8 Hz, 1H), 6.03-5.96 (m, 1H), 5.37-5.32 (m, 1H), 5.22-5.19 (m, 1H), 4.51-4.48 (m, 2H), 4.35 (s, 1H). HRMS: calculated for C_18_H_15_NO_2_ ([M+H]^+^) 278.1103, found 278.1109.

***N*-(3-(Allyloxy)phenyl)-1*H*-indazole-6-carboxamide (B5)**. White solid, 0.136 g, yield 46.9%. ^1^H NMR (600 MHz, DMSO) δ: 13.43 (s, 1H), 10.33 (s, 1H), 8.19 (s, 1H), 8.15 (s, 1H),7.90 (d, *J* = 8.4 Hz, 1H), 7.69-7.67 (m, 1H), 7.54 (t, *J* = 2.2 Hz, 1H), 7.41-7.39 (m, 1H), 7.27 (t, *J* = 8.1 Hz, 1H), 6.74-6.70 (m, 1H), 6.11-6.05 (m, 1H), 5.45-5.40 (dd, *J* = 17.3, 1.7 Hz, 1H), 5.30-5.27 (m, 1H), 4.58 (dt, *J* = 5.2, 1.5 Hz, 2H). ^13^C NMR (151 MHz, DMSO) δ: 166.3, 158.8, 140.8, 139.7, 134.1, 133.1, 129.8, 124.8, 120.9, 120.0, 117.8, 113.1, 110.4, 110.2, 107.3, 68.5. HRMS: calculated for C_17_H_15_N_3_O_2_ ([M+H]^+^) 294.1237, found 294.1252.

***N*-(3-(Allyloxy)phenyl)quinoline-3-carboxamide (B6)**. White solid, 0.075 g, yield 42%. ^1^H NMR (600 MHz, DMSO) δ: 10.59 (s, 1H), 9.37 (d, *J* = 2.2 Hz, 1H), 8.97 (d, *J* = 1.9 Hz, 1H), 8.16 (dd, *J* = 22.5, 8.2 Hz, 2H), 7.95-7.91 (m, 1H), 7.76 (t, *J* = 7.4 Hz, 1H), 7.56 (s, 1H), 7.41 (d, *J* = 18 Hz, 1H), 7.31 (t, *J* = 8.1 Hz, 1H), 6.77 (dd, *J* = 8.2, 2.3 Hz, 1H), 6.13-6.06 (m, 1H), 5.45 (dd, *J* = 17.3, 1.5 Hz, 1H), 5.32-5.29 (m, 1H), 4.61 (d, *J* = 5.1 Hz, 2H). ^13^C NMR (151 MHz, DMSO) δ: 164.6, 158.9, 149.5, 148.9, 140.5, 136.4, 134.1, 131.8, 129.9, 129.6, 129.2, 128.1, 128.0, 126.8, 117.9, 113.1, 110.6, 107.3, 68.6. HRMS: calculated for C_19_H_16_N_2_O_2_ ([M+H]^+^) 305.1290, found 305.1291.

***N*-(3-(Allyloxy)phenyl)quinoline-7-carboxamide (B7)**. Yellow solid, 0.098 g, yield 62.1%. ^1^H NMR (600 MHz, DMSO) δ: 10.53 (s, 1H), 9.03 (dd, *J* = 4.1, 1.6 Hz, 1H), 8.69 (s, 1H), 8.46 (d, *J* = 8.2 Hz, 1H), 8.15-8.09 (m, 2H), 7.67-7.63 (m, 1H), 7.60-7.56 (m, 1H), 7.45 (d, *J* = 8.1 Hz, 1H), 7.28 (t, *J* = 8.1 Hz, 1H), 6.73 (dd, *J* = 8.2, 2.4 Hz, 1H), 6.07 (ddd, *J* = 22.4, 10.5, 5.2 Hz, 1H), 5.42 (dd, *J* = 17.3, 1.7 Hz, 1H), 5.28 (dd, *J* = 10.5, 1.4 Hz, 1H), 4.58 (d, *J* = 5.2 Hz, 2H). HRMS: calculated for C_19_H_16_N_2_O_2_ ([M+H]^+^) 305.1212, found 305.1215.

***N*-(3-(Allyloxy)phenyl)-4-methoxybenzenesulfonamide (B8)**. Intermediate **4a** (0.081 g, 0.53 mmol), 4-methoxy benzenesulfyl chloride (0.1 g, 0.48 mmol) and pyridine (1mL) were dissolved in dichloromethane (DCM) and reacted at room temperature under the protection of nitrogen for 2 h. After the reaction, the organic phase was diluted with DCM (20 mL), and then washed with dilute HCl, saturated NaHCO_3_ and saturated NaCl solution successively. Finally, the organic phase was collected, dried with anhydrous Na_2_SO_4_, reduced pressure concentration and column chromatography to obtain target compound **B8** (0.062 g, yield 40.1%) as white solid. ^1^H NMR (600 MHz, DMSO) δ: 10.15 (s, 1H), 7.71 (d, *J* = 8.9 Hz, 2H), 7.11 (s, 1H), 7.07 (d, *J* = 8.9 Hz, 2H), 6.70-6.66 (m, 2H), 6.60 (dd, *J* = 8.2, 2.1 Hz, 1H), 6.02-5.95 (m, 1H), 5.35 (dd, *J* = 17.3, 1.6 Hz, 1H), 5.24 (dd, *J* = 10.5, 1.4 Hz, 1H), 4.46 (d, *J* = 5.2 Hz, 2H), 3.80 (s, 3H). HRMS: calculated for C_16_H_17_NO_4_S ([M+H]^+^) 320.0878, found 320.0884.

The synthetic methods of target compounds **B9** refer to compound **B8**.

***N*-(4-(Allyloxy)phenyl)quinoline-8-sulfonamide (B9)**. Brown solid, 0.10 g, yield 39.5%. ^1^H NMR (600 MHz, DMSO) δ: 10.05 (s, 1H), 9.17-9.15 (m, 1H), 8.54-8.51 (m, 1H), 8.39 (dd, *J* = 7.3, 1.2 Hz, 1H), 8.28 (dd, *J* = 8.2, 1.1 Hz, 1H), 7.75-7.72 (m, 2H), 6.98 (t, *J* = 18 Hz, 1H), 6.67-6.64 (m, 2H), 6.49 (dd, *J* = 8.3, 1.7 Hz, 1H), 5.95-5.88 (m, 1H), 5.29-5.25 (m, 1H), 5.20-5.17 (m, 1H), 4.37 (d, *J* = 5.3 Hz, 2H). ^13^C NMR (151 MHz, DMSO) δ: 158.7, 151.8, 143.1, 139.4, 137.4, 135.6, 134.7, 133.8, 132.6, 130.0, 128.8, 126.0, 123.0, 117.9, 112.3, 109.9, 106.7, 68.4. HRMS: calculated for C_18_H_16_N_2_O_3_S ([M+H]^+^) 341.0954, found 341.0962..

**4-Methoxyphenyl (3-(allyloxy)phenyl)carbamate (B10)**. Intermediate **4a** (0.14 g, 0.74 mmol) was dissolved in a mixed solution of ethyl acetate, tetrahydrofuran and water and stirred at 0 °C. Then compounds 4-methoxyphenyl chloroformate (0.11 g, 0.67 mmol) and sodium bicarbonate (0.043 g, 0.41 mmol) were added for reaction for 1 h. Then the reaction was carried out at room temperature. After the reaction, EtOAc (30 mL) was first used for extraction, then saturated NaCl solution (2 × 20 mL) was used for washing, organic phase was collected, dried over anhydrous Na_2_SO_4_, decompression concentration and column chromatography was performed to obtain target compound **B10** (0.036g, yield 26.7%) as black solid. ^1^H NMR (600 MHz, DMSO) δ: 10.15 (s, 1H), 7.22 (t, *J* = 16.8 Hz, 1H), 7.20 (s,1H), 7.17-7.14 (m, 2H), 7.08 (d, *J* = 8.2 Hz, 1H), 7.00-6.96 (m, 2H), 6.66 (dd, *J* = 8.1, 1.8 Hz, 1H), 6.08-6.01 (m, 1H), 5.41 (dd, *J* = 17.2, 1.8 Hz, 1H), 5.27 (dd, *J* = 10.5, 1.5 Hz, 1H), 4.56-4.52 (m, 2H), 3.78 (s, 3H). HRMS: calculated for C_19_H_16_N_2_O_2_ ([M+H]^+^) 300.1158, found 300.1163.

**1-(3-Hydroxyphenyl)-3-(quinolin-6-yl)urea** (**18**). 6-aminoquinoline (0.21 g, 1.4 mmol) was dissolved in DCM, and a mixed solution of 3-methoxyphenyl isocyanate (0.24 g, 1.4 mmol) and DCM was slowly added dropwise to the reaction solution and stirred for 1 h, the reaction solution was concentrated to obtain intermediate **17**. Intermediate **17** (0.41 g, 1.3 mmol) was dissolved in 33% hydrobromic acid/acetic acid solution and stirred at 70 °C for 2 h. After the reaction, 2 M sodium hydroxide solution was added to adjust the pH to be basic, followed by EtOAc (3 × 20 mL) was extracted. The organic phase was collected, dried over anhydrous Na_2_SO_4_, concentrated under reduced pressure and subjected to column chromatography to obtain intermediate **18** as black solid (0.28 g, yield 73.6%).

**1-(3-(Allyloxy)phenyl)-3-(quinolin-6-yl)urea (C1)**. Intermediate **18** (0.11 g, 0.36 mmol), allyl bromide (70 mg, 0.54 mmol), and potassium carbonate (99 mg, 0.72 mmol) were stirred in ethanol solvent for 2 h. The reaction mixture was filtered, and washed with EtOAc (20 mL), and the organic phase was concentrated and subjected to column chromatography to obtain target compound **C1** (0.042 g, yield 37%) as white solid. ^1^H NMR (600 MHz, DMSO) δ: 9.02 (s, 1H), 8.80 (s, 1H), 8.73 (dd, *J* = 4.2, 1.7 Hz, 1H), 8.26-8.22 (m, 1H), 8.16 (d, *J* = 2.4 Hz, 1H), 7.93 (d, *J* = 9.0 Hz, 1H), 7.68 (dd, *J* = 9.1, 2.4 Hz, 1H), 7.44 (dd, *J* = 8.3, 4.2 Hz, 1H), 7.25 (t, *J* = 2.2 Hz, 1H), 7.18 (t, *J* = 8.1 Hz, 1H), 6.97-6.93 (m, 1H), 6.58 (ddd, *J* = 8.2, 2.5, 0.8 Hz, 1H), 6.11-5.97 (m, 1H), 5.42-5.37 (m, 1H), 5.27-5.24 (m, 1H), 4.54 (dt, *J* = 5.2, 1.6 Hz, 2H). HRMS: calculated for C_19_H_17_N_3_O_2_ ([M+H]^+^) 320.1321, found 320.1331.

**Methyl quinoxaline-6-carboxylate (20)**. 6-Quinoxalinecarboxylic acid (1.1 g, 5.74 mmol) and thionyl chloride (1.0 mL) were stirred in methanol (10 mL) at reflux for 2 h. After the reaction, water (30 mL) was added for quenching, EtOAc (3 × 30 mL) was used for extraction. The organic phase was collected, dried over anhydrous Na_2_SO_4_, concentrated and subjected to column chromatography to obtain intermediate **20** (1.0 g, yield 95%) as yellow oil.

**Quinoxaline-6-carbohydrazide (21)**. Intermediate **20** (0.30 g, 1.6 mmol) and hydrazinium hydroxide solution (1.2 g, 24 mmol) were stirred in methanol at reflux for 2h. After the reaction, the methanol was spin-dried, 2 M dilute HCl was added to adjust the pH to neutral and extracted with EtOAc (2 × 30 mL). The organic phase was collected, dried over anhydrous Na_2_SO_4_, concentrated and subjected to column chromatography to obtain intermediate **21** (0.19 g, yield 63.2%) as white oil.

**(*E*)-*N'*-(3-(Allyloxy)benzylidene)quinoxaline-6-carbohydrazide (C2)**. Intermediate **21** (0.050 g, 0.27 mmol) and 3-(allyloxy)benzaldehyde (0.055 g, 0.29 mmol) were dissolved in methanol, and acetic acid were added to react at room temperature for 2 h. After the reaction, water (20 mL) was added to dilute, and then extracted with EtOAc (2 × 20 mL). The organic phase was collected, dried over anhydrous Na_2_SO_4_, concentrated and subjected to column chromatography to obtain the target product **C2** (0.064 g, yield 72.3%) as white solid. ^1^H NMR (600 MHz, DMSO) δ: 12.23 (s, 1H), 9.06 (dd, *J* = 8.4, 1.7 Hz, 2H), 8.70 (d, *J* = 1.8 Hz, 1H), 8.48 (s, 1H), 8.32 (dd, *J* = 8.7, 1.9 Hz, 1H), 8.24 (d, *J* = 8.7 Hz, 1H), 7.39 (t, *J* = 8.0 Hz, 1H), 7.33 (d, *J* = 7.5 Hz, 2H), 7.07*-*7.02 (m, 1H), 6.07 (ddd, *J* = 22.4, 10.4, 5.2 Hz, 1H), 5.43 (dd, *J* = 17.3, 1.7 Hz, 1H), 5.28 (dd, *J* = 10.6, 1.4 Hz, 1H), 4.66*-*4.60 (m, 2H).^13^C NMR (151 MHz, DMSO) δ: 162.5, 158.9, 148.8, 147.6, 147.2, 144.0, 142.0, 136.1, 134.9, 134.0, 130.4, 130.0, 129.3, 129.1, 120.6, 117.9, 117.4, 112.9, 68.7. HRMS: calculated for C_19_H_16_N_4_O_2_ ([M+H]^+^) 333.1346, found 333.1359.

The synthetic methods of target compounds **C3** refer to compound **C2**.

**(*E*)-*N'*-(4-(Allyloxy)benzylidene)quinoxaline-6-carbohydrazide (C3)**. White solid, 0.066 g, yield 74.5%. ^1^H NMR (600 MHz, DMSO) δ: 12.09 (s, 1H), 9.05 (dd, *J* = 8.9, 1.7 Hz, 2H), 8.69 (d, *J* = 1.8 Hz, 1H), 8.46 (s, 1H), 8.31 (dd, *J* = 8.7, 1.9 Hz, 1H), 8.23 (d, *J* = 8.7 Hz, 1H), 7.70 (d, *J* = 8.8 Hz, 2H), 7.05 (d, *J* = 8.8 Hz, 2H), 6.09-6.02 (m, 1H), 5.41 (dd, *J* = 17.3, 1.7 Hz, 1H), 5.28 (dd, *J* = 10.5, 1.5 Hz, 1H), 4.63 (dd, *J* = 3.8, 1.4 Hz, 2H). ^13^C NMR (151 MHz, DMSO) δ: 166.3, 158.8, 140.8, 139.7, 134.1, 133.1, 129.8, 124.8, 120.9, 120.0, 117.8, 113.1, 110.4, 110.2, 107.3, 68.5. HRMS: calculated for C_19_H_16_N_4_O_2_ ([M+H]^+^) 333.1346, found 333.1358.

**2-(3-(Cyclopropylmethoxy)phenoxy)-*N*-(quinoxalin-6-yl)acetamide (C4)**. Resorcinol (0.51 g, 3.59 mmol) and bromomethyl cyclopropane (0.73 g, 5.4 mmol), potassium carbonate (0.99 g, 7.18 mmol) were reacted in acetone at reflux for 2 h. After the reaction, filtered, the filter cake was washed with EtOAc (30 mL). The organic phase was collected, concentrated under reduced pressure and subjected to column chromatography to obtain intermediate **25** as pale red oil. 6-Aminoquinoline (1.1 g, 6.9 mmol) and *N*,*N*-diisopropylethylamine (1.1 g, 8.6 mmol) were dissolved in THF, then chloroacetyl chloride (0.78 g, 6.9 mmol) dropwise ) and tetrahydrofuran were added, the reaction was stirred at 0 °C with for 1 h. After the reaction, the water (50 mL) was added for quenching, EtOAc (3 × 20 mL) was used for extraction. The organic phase was collected, dried over anhydrous Na_2_SO_4_, concentrated and subjected to column chromatography to give intermediate **27** as yellow solid. Finally, intermediate **25** (0.10 g, 0.45 mmol), intermediate **27** (0.10 g, 0.68 mmol) and potassium carbonate (0.19 g, 1.4 mmol) were stirred at reflux in ethanol for 4-5 h. After the reaction, filtered, washed the filter cake with EtOAc (2 × 20 mL), collected the organic phase, concentrated under reduced pressure and subjected to column chromatography to obtain the target compound **C4** (0.087 g, yield 78.5%） as yellow solid. ^1^H NMR (300 MHz, DMSO) δ: 10.60 (s, 1H), 8.90 (d, *J* = 1.8 Hz, 1H), 8.85 (d, *J* = 1.8 Hz, 1H), 8.56 (d, *J* = 2.3 Hz, 1H), 8.09 (d, *J* = 9.1 Hz, 1H), 8.04 (dd, *J* = 9.1, 2.3 Hz, 1H), 7.24*-*7.18 (m, 1H), 6.63*-*6.59 (m, 2H), 6.58*-*6.55 (m, 1H), 4.80 (s, 2H), 3.81 (d, *J* = 7.0 Hz, 2H), 1.24 (s, 1H), 0.58*-*0.53 (m, 2H), 0.33*-*0.28 (m, 2H). HRMS: calculated for C_20_H_19_N_3_O_3_ ([M+H]^+^) 350.1426, found 350.1429.

# *In Vitro* Cell Cytotoxicity Evaluation

Human normal cells (HUVEC) in better adherent condition were collected and resuspended in culture medium DMEM and maintained at a cell concentration (5 × 10^4^ CFU/mL) was added to columns 2-11 in 96-well plates (3 × 100 μL) , incubated in a CO_2_ incubator. After attachment of the cells, the medium was discarded, and the test compound solution (3 × 100 μL) was added to columns 2-10 in 96-well plates. Only the cell culture medium was added in the 11th column as a positive control, and the 12th column was only added PBS was used as a negative control. After 48 h of incubation, the medium of each well was discarded and freshly prepared 10% CCK-8 solution (90 μL basal medium + 10 μL CCK-8) was added in the dark, and the cells continue to be incubated at 37 °C for 30 min to ensure that all the cells were stained. The absorbance A at 450 nm of each well in a 96 well plate was detected, according to the formula: inhibition rate% = (A _positive control well_-A _compound well_) / (A _positive control well_-A _negative control well_) × 100%, IC_50_ values were calculated.

# Spectral Data

^1^H NMR (600 MHz) and ^13^C NMR (150 MHz) of compound **A1** in DMSO-*d_6_*


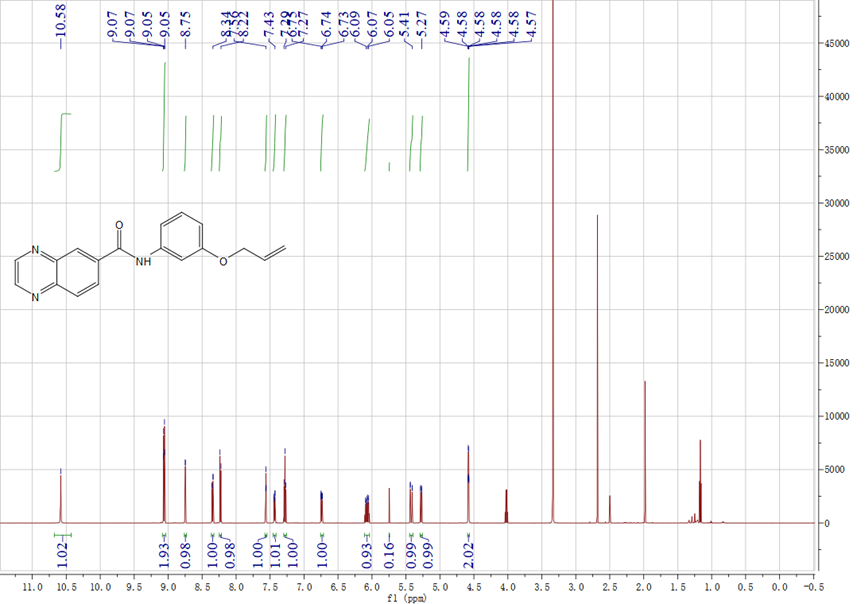


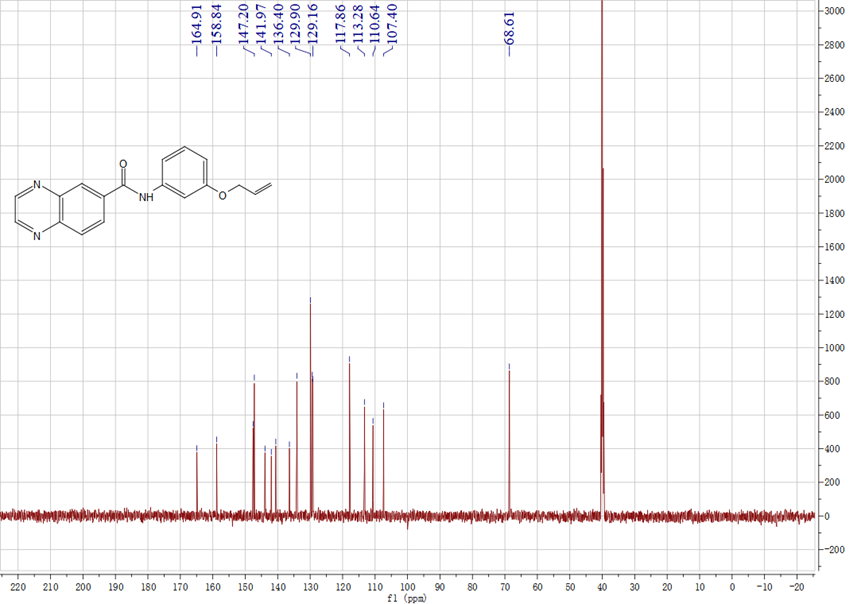


^1^H NMR (600 MHz) and ^13^C NMR (150 MHz) of compound **A2** in DMSO-*d_6_*


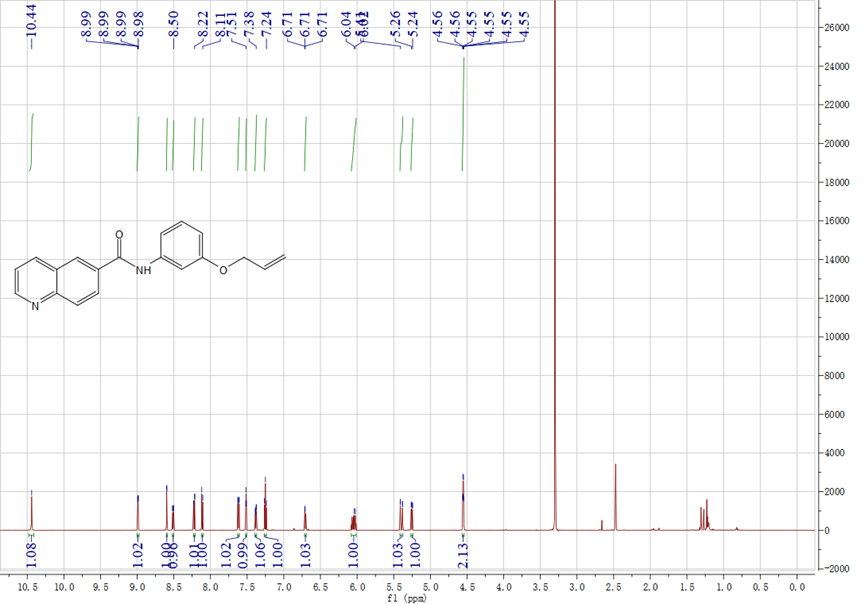


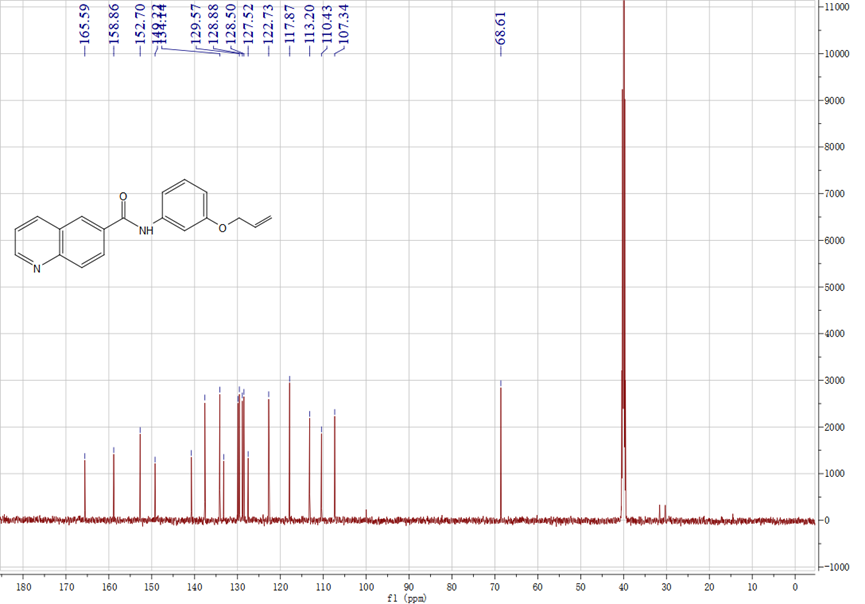


ESI-HRMS of compound **A1**


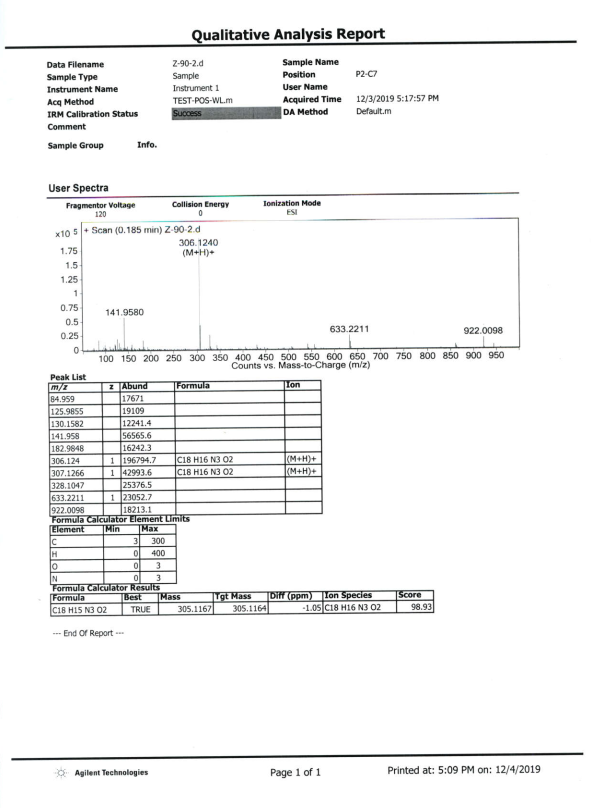


ESI-HRMS of compound **A2**


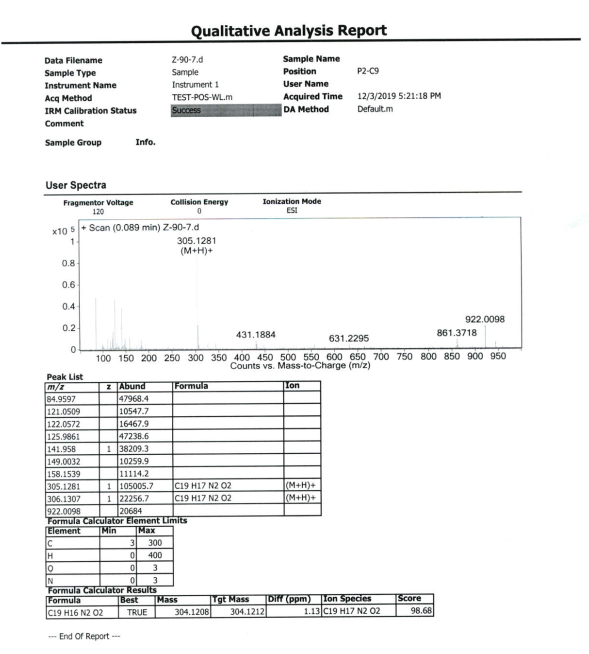

Supplement: Supplemental Material [file TEMI_A_2208687_SM3476.docx]
